# Identification, Classification and Differential Expression of Oleosin Genes in Tung Tree (*Vernicia fordii*)

**DOI:** 10.1371/journal.pone.0088409

**Published:** 2014-02-06

**Authors:** Heping Cao, Lin Zhang, Xiaofeng Tan, Hongxu Long, Jay M. Shockey

**Affiliations:** 1 U.S. Department of Agriculture, Agricultural Research Service, Southern Regional Research Center, Commodity Utilization Research Unit, New Orleans, Louisiana, United States of America; 2 Key Laboratory of Cultivation and Protection for Non-Wood Forest Trees, Ministry of Education, Central South University of Forestry and Technology, Changsha, Hunan Province, People's Republic of China; University Paris South, France

## Abstract

Triacylglycerols (TAG) are the major molecules of energy storage in eukaryotes. TAG are packed in subcellular structures called oil bodies or lipid droplets. Oleosins (OLE) are the major proteins in plant oil bodies. Multiple isoforms of OLE are present in plants such as tung tree (*Vernicia fordii*), whose seeds are rich in novel TAG with a wide range of industrial applications. The objectives of this study were to identify OLE genes, classify OLE proteins and analyze OLE gene expression in tung trees. We identified five tung tree OLE genes coding for small hydrophobic proteins. Genome-wide phylogenetic analysis and multiple sequence alignment demonstrated that the five tung OLE genes represented the five OLE subfamilies and all contained the “proline knot” motif (PX5SPX3P) shared among 65 OLE from 19 tree species, including the sequenced genomes of *Prunus persica* (peach), *Populus trichocarpa* (poplar), *Ricinus communis* (castor bean), *Theobroma cacao* (cacao) and *Vitis vinifera* (grapevine). Tung OLE1, OLE2 and OLE3 belong to the S type and OLE4 and OLE5 belong to the SM type of *Arabidopsis* OLE. TaqMan and SYBR Green qPCR methods were used to study the differential expression of OLE genes in tung tree tissues. Expression results demonstrated that 1) All five OLE genes were expressed in developing tung seeds, leaves and flowers; 2) OLE mRNA levels were much higher in seeds than leaves or flowers; 3) OLE1, OLE2 and OLE3 genes were expressed in tung seeds at much higher levels than OLE4 and OLE5 genes; 4) OLE mRNA levels rapidly increased during seed development; and 5) OLE gene expression was well-coordinated with tung oil accumulation in the seeds. These results suggest that tung OLE genes 1–3 probably play major roles in tung oil accumulation and/or oil body development. Therefore, they might be preferred targets for tung oil engineering in transgenic plants.

## Introduction

Tung tree (*Vernicia fordii*) is an economically important tree with a very limited growing area in the United States [1], [2]. Tung seeds contain approximately 50–60% oil (dry weight basis) with about 80 mole % α-eleostearic acid (9*cis*, 11*trans*, 13*trans* octadecatrienoic acid) [3]. Tung oil is readily oxidized because of the three unique conjugated double bonds in eleostearic acid. Dried tung oil is impervious to heat, moisture, dust and many chemical challenges. Tung oil, unlike other drying oils, does not darken with age. These properties of tung oil make it a widely used drying ingredient in paints, varnishes, coatings and finishes [4], [5]. Recently, tung oil has been explored as a raw material to produce biodiesel [6]–[8], polyurethane and wood flour composites [9], thermosetting polymer [10] and repairing agent for self-healing epoxy coatings [11].

Our project focuses on alternative ways of producing tung oil-like fatty acids and other high-value industrial oils by engineering tung oil biosynthetic genes into oilseed crops. Many tung oil biosynthetic genes have been identified in our laboratories, including those coding for diacylglycerol acyltransferases (DGAT) [12], [13], delta-12 oleic acid desaturase (FAD2) and delta-12 fatty acid conjugase (FADX) [14], omega-3 fatty acid desaturase (FAD3) [15], acyl-CoA binding proteins [16], cytochrome b5 [17], cytochrome b5 reductase [18], glycerol-3-phosphate acyltransferase (GPAT) [19], plastid-type omega-3 fatty acid desaturase (TnDES2) [20], aquaporin [21] and glutaredoxin [21]. However, selection of target genes for genetic engineering of plant oils is difficult because oil is biosynthesized by at least 10 enzymatic steps and each step is catalyzed by multiple isozymes [22]–[24]. Furthermore, it has been difficult to study tung oil biosynthesis at the protein level because these enzymes are mostly hydrophobic and membrane-localized proteins [25], [26].

Triacylglycerols (TAG) such as tung oil accumulate in discrete subcellular structures called oil bodies in plants, similar to oil droplets in animals. Plant oil bodies mainly consist of TAG surrounded by a monolayer of phospholipids, with the hydrophobic acyl moieties of the phospholipids interacting with TAG and the hydrophilic head groups facing the cytosol [27], [28]. In addition, plant oil bodies contain a number of proteins including oleosins (OLE) and caleosins [27], [29], [30].

OLE are a group of hydrophobic proteins localized on the surfaces of plant oil bodies founded primarily in the seeds and pollen. The precise functions of OLE are unknown. They may function to stabilize oil bodies at low water potential and/or regulate the sizes of oil bodies [31]. Recent research suggests that OLE may be bifunctional enzymes with both monoacylglycerol acyltransferase and phospholipase activities regulated by serine/threonine/tyrosine protein kinases [32], [33]. The tung genome is known to contain multiple oleosin genes [34], [35] but the oleosin protein makeup of tung seed oil bodies is unknown. A number of OLE EST sequences from tung tree have been deposited in the GenBank database [35]. Two tung tree OLE cDNA clones were described in a preliminary report published by a Chinese journal [36]. However, no data is available on the expression of any OLE gene in tung tree [35], [36].

The objectives of this study were to identify OLE genes, classify OLE proteins and analyze OLE gene expression in tung trees. We identified five OLE genes in tung tree. We performed genome-wide phylogenetic analysis and multiple sequence alignment and classified the five tung OLE genes based on 65 OLE from 19 tree species including the sequenced genomes of *Prunus persica* (peach) [37], *Populus trichocarpa* (poplar) [38], *Ricinus communis* (castor bean) [39], *Theobroma cacao* (cacao) [40] and *Vitis vinifera* (grapevine) [41]. We also classified tung OLE according to the well-known 17 subfamilies of *Arabidopsis* OLE. Finally, we used TaqMan and SYBR Green qPCR assays to evaluate the relative abundance and tissue distribution of the five OLE mRNA in the seeds, leaves and flowers of tung trees.

## Materials and Methods

### Plant Materials

Tung trees were grown in the American Tung Oil Corporation orchard in Lumberton, Mississippi. John Corley, the Company officer, granted permission for this field study. Tung fruits were collected weekly for 11 weeks beginning June 23, 2006 (week 1). The developmental stage of week 1 seeds corresponded to approximately 9 weeks after full bloom and 1 month before the initiation of storage oil synthesis. Tung tree seeds were removed from the trees and kernels and immediately frozen in liquid N_2_ and stored at −80°C. The oil and fatty acid profiles of these tung seeds were reported previously by Cao et al, 2013 [13].

### Identification of Oleosin Genes in Tung Tree

The tung seed cDNA library used for EST analysis was constructed previously [14] using the TriplEx system (Clontech, Mountain View, CA, USA). Initial gene discovery was derived from random sequencing of a plasmid-based tung seed cDNA library, as described previously [14]. Additional gene discovery was enabled through 454 pyrosequencing of cDNA samples from developing tung seeds, created from reverse transcription of RNA samples extracted using the method of Wan and Wikins, as described previously [12], [42]. Normalized and non-normalized 454 samples were prepared as described previously [16].

### Computational Methods

OLE sequences from other organisms were obtained from database searches using the keyword “oleosin” and BlastP searches [43], [44] against the National Center for Biotechnology Information (NCBI)'s non-redundant protein sequence databases (http://blast.ncbi.nlm.nih.gov/Blast.cgi) using tung tree OLE sequences. The properties and amino acid compositions of OLE were analyzed using Vector NTI software (Life Technologies, Carlsbad, CA) [45]. Statistics were performed using Microsoft Excel. Phylogenetic analysis for studying the presumed evolutionary relationships among the OLE proteins was performed using the Vector NTI software based on the neighbor-joining method of Saitou and Nei [46]. Multiple sequence alignment was performed using the ClustalW algorithm [47], [48] of the AlignX program of the Vector NTI software. This method is based on algorithms that assign scores to aligned residues and detect sequence similarities. Identical amino acid residues in alignment have higher scores than those not identical and less similar residues.

### RNA Isolation

Total RNAs from tung seeds, leaves and flowers were isolated as described by Spectrum Plant Total RNA Kit (Sigma) [49] and the hot borate method [42]. RNA concentrations and integrity were determined using RNA 6000 Nano Assay Kit and the Bioanalyzer 2100 (Agilent Technologies) with RNA 6000 Ladder as the standards [50]. The RNAs isolated from tung tissues were high quality because the RNA preparations have high rRNA ratio (28S/18S = 1.9) and the RNA integrity number (RIN = 8.7) [49].

### cDNA Synthesis

The cDNAs were synthesized from total RNAs using SuperScript II reverse transcriptase as described [49]. The cDNA synthesis mixture in 20 µl contained 5 µg total RNA, 2.4 µg oligo(dT)_12–18_ primer, 0.1 µg random primers, 500 µM dNTPs, 10 mM DTT, 40 u RNaseOUT, and 200 u SuperScript II reverse transcriptase in 1X first-strand synthesis buffer (Life Technologies, Carlsbad, CA). The cDNA synthesis reaction was kept at 42°C for 50 min. The cDNAs were stored in −80°C freezer before qPCR analyses.

### qPCR Primers and Probes

PCR primers and TaqMan probes were designed using Primer Express software (Applied Biosystems, Foster City, CA, USA). The *T*_m_s for the probes were approximately 10°C higher than the corresponding primers. They were synthesized by Biosearch Technologies, Inc (Navato, CA, USA). The amplicon sizes and the nucleotide sequences (5′ to 3′) of the forward primers, TaqMan probes (TET–BHQ1) and reverse primers of OLE are described in Table 1. The reference genes coding for tung 60 s ribosome protein L19 (Rpl19b), ubiquitin protein ligase (Ubl) and glyceraldehyde 3 phosphate dehydrogenase (Gapdh) were described [51]. Tung Rpl19b and Ubl are preferable to Gapdh as the best reference genes for both TaqMan and SYBR Green qPCR assays for quantitative gene expression analysis in tung trees [52].

**Table 1:** Ole gene expression profiles analyzed by qPCR and the nucleotide sequences of real-time PCR primers and TaqMan probes.

mRNA	Name	Accession number	Amplicon	Forward primer (5′ to 3′)	TaqMan probe (5′ to 3′)	Reverse primer (5′ to 3′)
Ole1	Oleosin 1	GU245884	59 bp	AAGGCACGGGAAATGAAAGA	AGGGCTGAGCAGTTAG	TGTTGGCCCGTTACATGCT
Ole2	Oleosin 2	GU245885	56 bp	GAGGCCACTCGGAACATACC	AGCAGCTGGATCAGG	TCTTGCATGCGCCTCCTT
Ole3	Oleosin 3	GR217754	57 bp	TGCACGCGCCGCTTA	CATGTTCCATCTGCAGCG	CCGTAGGATGAGAGGCTCTTTG
Ole4	Oleosin 4	This report	61 bp	GGCGGTTGTGGGTGGAT	ATCTTGGGCTTATAGGTATT	ACCGGGTGGATTCATACCTCTA
Ole5	Oleosin 5	GR218198	57 bp	CTGTGCCTTTTTCGCAATTTT	CCTCTCGCATAATC	CCGCCTGGTGCTGATAAGTT

### qPCR Assays

The optimized qPCR reaction mixtures contained variable amounts of total RNA-derived cDNA (2.5, 5, 12.5, and 25 ng), 200 nM each of the forward primer, reverse primer, and TaqMan probe and 1× Absolute QPCR Mix (ABgene House, Epson, Surrey, UK) (TaqMan qPCR) or 1 x iQ SYBR Green Supermix (Bio-Rad Laboratories) without the TaqMan probes (SYBR Green qPCR) [50]. The reactions were performed in 96-well clear plates sealed by adhesives with a CFX96 real-time system-C1000 Thermal Cycler (Bio-Rad Laboratories). The thermal cycle conditions for TaqMan assay were as follows: 2 min at 50°C and 15 min at 95°C (This step is required for the activation of Thermo-Start DNA polymerase), followed by 50 cycles at 95°C for 15 s and 60°C for 60 s. The thermal cycle conditions for SYBR Green assay were as follows: 3 min at 95°C, followed by 40 cycles at 95°C for 10 s, 65°C for 30 s and 72°C for 30 s. Agarose gel electrophoresis was used to confirm the specificity of qPCR amplification using 3% agarose gel for separating qPCR products at 100 V for 30 min as shown previously [13].

### Data Analysis

The ΔΔC_T_ method of relative quantification was used to determine the fold change in expression [53]. This was done by first normalizing the threshold cycle (C_T_) values of the target mRNAs to the C_T_ values of the internal reference mRNA Rpl19b, Gapdh or Ubl in the same samples (ΔC_T_ = C_TTarget_−C_Tref_). The ΔC_T_ was further normalized with a calibrator, the sample control (ΔΔC_T_). The fold change in expression was then obtained (2^−ΔΔCT^). The amplification efficiency of qPCR assay was estimated on the basis of the equation *E* = (10^−1/slope^−1)×100 [54]. The means and standard deviations presented in the tables and figures were determined from 4–6 assays for each mRNA.

## Results

### Identification of OLE Genes in Tung Tree

**Figure 1:**
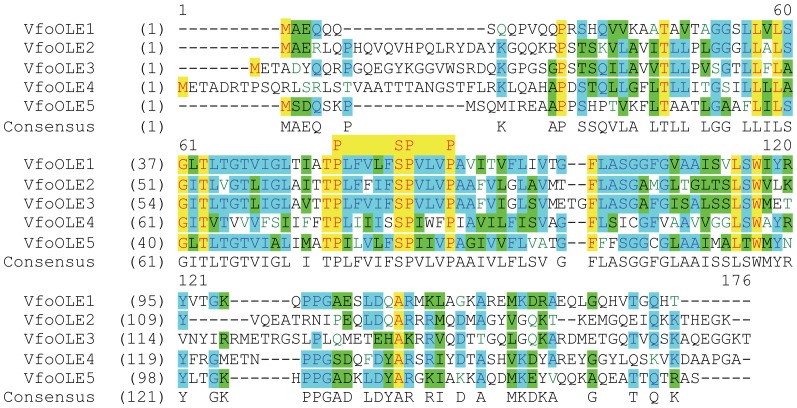
Amino acid sequence alignment of the five tung tree OLE proteins. Multiple sequence alignment was performed using the ClustalW algorithm of the AlignX program of the Vector NTI software. OLE name is on the left of alignment followed by the start of the amino acid sequence of each OLE protein. The numbers at the top of the alignment are the positions of the multiple sequence alignment. The letters at the bottom of the alignment are the consensus amino acid residues. Residues in red on yellow represent those conserved in all five OLE sequences at a given position, whereas those in black on blue represent residues conserved in majority of the sequences at a given position.

Anonymous tung seed cDNA sequences from week 6 were generated and analyzed by pyrosequencing (“454”) technology as described previously [16]. Multiple forms of OLE genes were found in these U.S. samples. Four of them corresponded to the sequences identified in the Chinese samples, which were deposited in the Genbank database (Table 1). The other tung oleosin in U.S. samples (named OLE4) shared significant conservation with the four tung OLE at the protein and nucleotide levels; 16 amino acid residues out of 169 and 123 nucleotides out of 820 were completely conserved among the five tung OLE at the protein and DNA levels, respectively (Figure 1 and Figure S1). The signature sequences of OLE called “proline knot” (PX5SPX3P) were completely conserved among the five tung tree OLE (Figure 1). These five forms of OLE were all small proteins with an average of 154 amino acid residues and calculated molecular mass of 16.5 kDa (Table 2). These proteins possessed high isoelectric point (9.35) and high percentage of hydrophobic resides (40.45%) (Table 2).

**Table 2:** The properties and amino acid composition of the tung Ole proteins.

Properties and amino acid composition (% by frequency)	OLE1	OLE2	OLE3	OLE4	OLE5	Mean ± SD
Length (amino acid residue)	137	154	169	169	142	154±11
Molecular weight (Da)	14409	16788	17964	18289	15106	16511±1269
Isoelectric point (PI)	9.87	9.93	8.25	9.17	9.52	9.35±0.62
Polar (NCQSTY) (%)	26.28	24.03	28.40	27.22	23.94	25.97±1.96
Hydrophobic (AILFWV) (%)	42.34	38.96	34.32	44.38	42.25	40.45±3.80

### Classification of Tung Tree OLE

**Figure 2:**
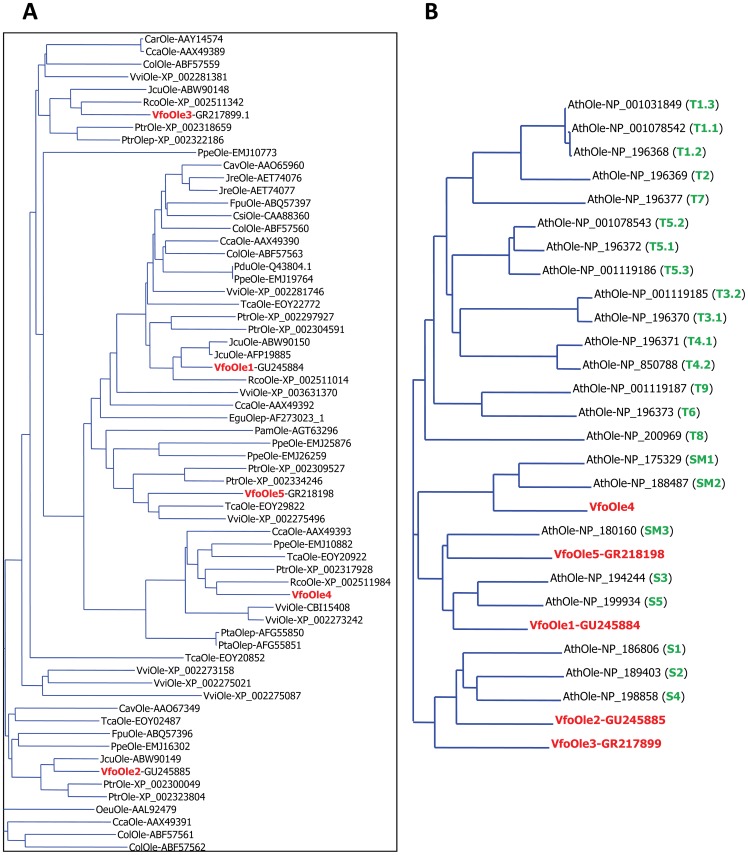
Phylogenetic analysis of 65 OLE from 19 tree species and 23 reference OLE from *Arabidopsis*. (A) Phylogenetic analysis of OLE from tung tree and other tree species. (B) Phylogenetic analysis of OLE from tung tree and *Arabidopsis*. Tung OLE are highlighted in red. The names of 17 subfamilies from 23 *Arabidopsis* OLE are highlighted in green. S, seed-specific OLE, SM, seed-microspore-specific OLE, T, tapetum-specific OLE. The abbreviations of the organisms are: Ath, *Arabidopsis thaliana*; Car, *Coffea arabica* (coffee); Cca, *Coffea canephora* (coffee); Cav, *Corylus avellana* (hazelnut); Col, *Camellia oleifera* (tea oil); Csi, *Citrus sinensis* (orange); Egu, *Elaeis guineensis* (oil palm); Fpu, *Ficus pumila* (climbing fig); Jcu, *Jatropha curcas* (barbados nut); Jre, *Juglans regia* (walnut); Oeu, *Olea europaea* (olive); Pam, *Persea Americana* (avocado); Pdu, *Prunus dulcis* (almond); Ppe, *Prunus persica* (peach); Pta, *Pinus taeda* (loblolly pine); Ptr, *Populus trichocarpa* (poplar); Rco, *Ricinus communis* (castor bean); Tca, *Theobroma cacao* (cacao); Vfo, *Vernicia fordii* (tung tree); Vvi, *Vitis vinifera* (grapevine).

GenBank database search identified 75 unique OLE from 22 trees. These OLE include 1 from *Coffea arabica* (coffee) [55], 5 from *Coffea canephora* (coffee) [55], 2 from *Corylus avellana* (hazelnut) [56], 3 from *Cocos nucifera* (coconut palm) [57], 5 from *Camellia oleifera* (tea oil), 5 from *Cupressus sempervirens* (pencil pine) [58], 1 from *Citrus sinensis* (orange) [59], 1 from *Elaeis guineensis* (oil palm) [60], 2 from *Ficus pumila* (climbing fig) [61], 5 from *Jatropha curcas* (barbados nut) [62]–[64], 1 from *Juglans regia* (walnut), 1 from *Olea europaea* (olive) [65], 1 from *Persea americana* (avocado) [66], 1 from *Prunus armeniaca* (apricot) [67], 1 from *Prunus dulcis* (almond) [68], 6 from *Prunus persica* (peach) [37], 2 from *Pinus taeda* (loblolly pine) [69], 9 from *Populus trichocarpa* (poplar) [38], 5 from *Ricinus communis* (castor bean) [39], 6 from *Theobroma cacao* (cacao) [40] and 9 from *Vitis vinifera* (grapevine) [41]. Sixty-five of these sequences from 19 tree species including five identified in this report contained the perfect “proline knot” motif (PX5SPX3P) (Figure S2). Phylogenic analysis showed that tung OLE were most closely related to castor bean OLE (Figure 2A). The five tung OLE represented the five subfamilies of OLE from diverse tree species (Figure 2A). Tung OLE contained all four invariant residues in the “proline knot” motif (PX5SPX3P) (Figure S2). In addition, the amino and carboxyl termini of tung OLE were properly aligned with those of the other plant OLE (Figure S2). These sequence analyses clearly support our conclusion that the identified OLE from tung tree are full-length.

*Arabidopsis* contains 17 subfamilies of OLE including 5 forms of seed-specific OLE (S type), 3 forms of seed-and-microspore-specific OLE (SM type) and 9 forms of tapetum-specific OLE (T type) [70]–[72]. Twenty-three reference genes code for OLE in *Arabidopsis* genome [73]–[79]. Tung OLE aligned with S and SM types but none of them aligned with T type of *Arabidopsis* OLE (Figure 2B). Tung OLE1 closely aligned with *Arabidopsis* S3 and S5 OLE, whereas tung OLE2 and OLE3 aligned with *Arabidopsis* S1, S2 and S4 OLE (Figure 2B). Tung OLE4 aligned with *Arabidopsis* SM1 and SM2 OLE and tung OLE5 aligned with *Arabidopsis* SM3 OLE (Figure 2B).

### Optimization, Specificity and Efficiency of qPCR Assay for OLE Gene Expression

**Figure 3:**
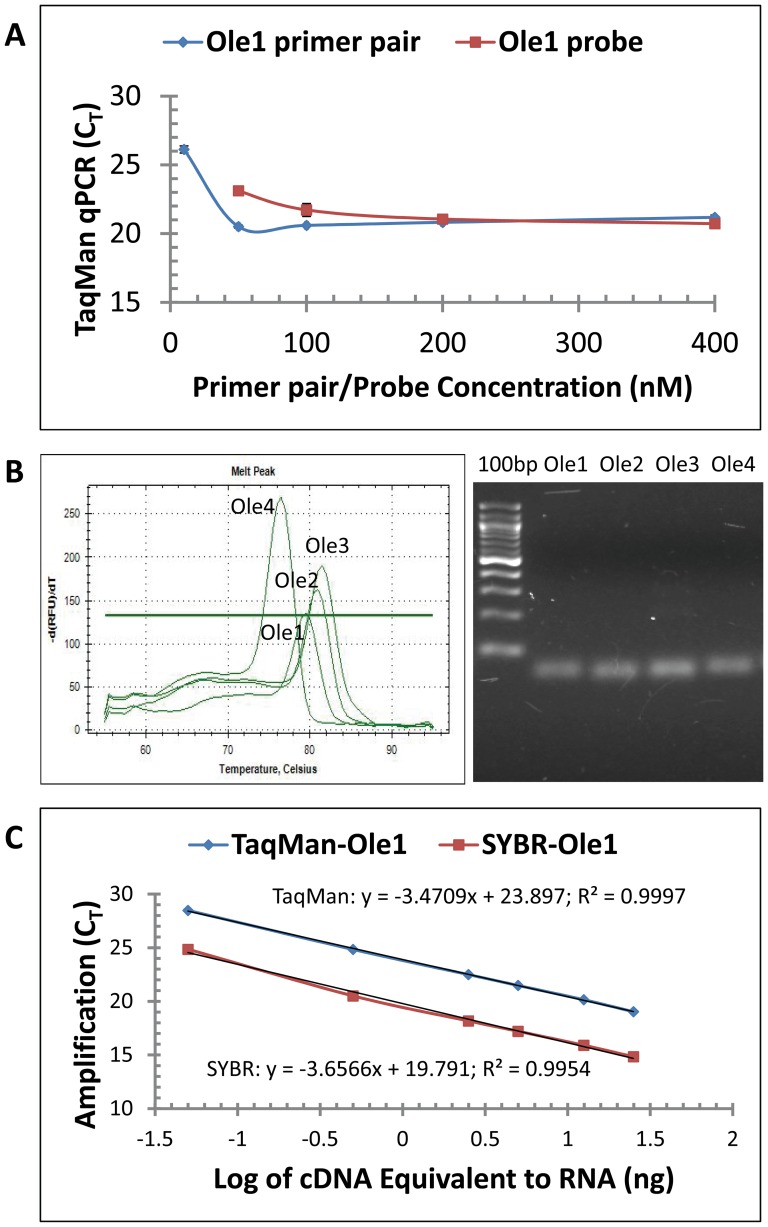
qPCR optimization, specificity and efficiency for OLE assay. (A) TaqMan qPCR optimization. TaqMan qPCR reactions contained 5 ng RNA-equivalent cDNA from tung seeds, various concentrations of the primers and TaqMan probe. Ole1 assay optimization is presented. (B) Specificity of SYBR Green qPCR by melt curve analysis and gel electrophoresis of amplification products. The qPCR reactions contained 5 ng RNA-equivalent cDNA from tung tree seeds. The qPCR products were separated by agarose gel electrophoresis. Lane 100 bp represents DNA ladders with 100 bp as the smallest band, increasing upward in 100 bp increments. The results using RNA isolated from leaves and flowers are presented in Figure S3. (C) qPCR efficiency for OLE assay. TaqMan and SYBR Green qPCR reaction mixtures contained variable concentrations of RNA-equivalent cDNA from tung seeds, the optimized concentrations of each primer and probe (200 nM), and Absolute QPCR Mix (TaqMan qPCR) or each primer and 1 x iQ SYBR Green Supermix (SYBR Green qPCR). The results using RNA isolated from stage 4 seeds of tree 1 are shown in the figure. The results for Ole2, Ole3 and Ole4 assays are presented in Figure S4. The results using RNA from other stages of tung seeds, leaves and flowers are presented in Table S1.

TaqMan and SYBR Green qPCR assays are widely used for quantitative analysis of gene expression. Figure 3A shows an example of primer and probe optimization for OLE1 mRNA quantification. Two hundred nM primer pair and probe concentrations saturated the TaqMan qPCR reactions. We thus used 200 nM for each of the primers and probes in the following qPCR assays. SYBR Green qPCR may generate false positive signals if nonspecific products or primer-dimers are present in the assay because the dye binds to all double-stranded DNA. Melt curve analysis shows that each OLE gene-specific qPCR resulted in a single peak of PCR amplification signal using total cDNA from seeds, leaves and flowers (Figure 3B and Figure S3). Agarose gel electrophoresis shows that amplification from each OLE gene resulted in a single DNA fragment matching the predicted size of the amplicon (Figure 3B and Figure S3). Both analyses indicated that SYBR Green qPCR assays were reliable for evaluating OLE family gene expression. Both qPCR assays generated similar slopes and correlation co-efficiencies but the TaqMan assays generated higher y-intercepts (Figure 3C and Figure S4). The TaqMan qPCR assay was selected for further analysis of qPCR efficiency using RNA from other stages of tung seeds and leaves and flowers. TaqMan qPCR generated high correlation co-efficiency (r^2^>0.99 in most assays) and good amplification efficiency for all cDNA samples (Table S1).

### Variations of OLE Gene Expression among Tung Trees

The development of tung trees varies significantly in terms of flowering time, which affects gene expression levels, seed development and oil accumulation in later stages. These variations affect data calculation using multiple tung trees. We therefore examined the variations of OLE gene expression among the tung trees using cDNA from multiple stages of developing tung seeds under optimized primer pair and probe concentrations. TaqMan qPCR shows that OLE1 mRNA levels in the seeds had the least variations among the trees with a mean and standard deviation of 0.90±0.09, whereas those of OLE2, OLE3 and OLE4 mRNA were 0.81±0.24, 0.80±0.35 and 0.75±0.24, respectively (Table 3). The variation of OLE5 gene expression was similar to that of OLE4 (data not shown). Similar results were obtained by SYBR Green qPCR assays (Table S2). These variations of OLE gene expression among the trees, in agreement with previous data on DGAT family gene expression [13], illustrate the difficulties of averaging data among the three trees and therefore data from tree 1 are presented in the following experiments.

**Table 3:** Variation of Ole gene expression among tung trees.

mRNA	Tree 1	Tree 2	Tree 3	Mean ± SD
	(fold)	(fold)	(fold)	(fold)
Ole1	1	0.89±0.27	0.81±0.40	0.90±0.09
Ole2	1	0.89±0.27	0.55±0.27	0.81±0.24
Ole3	1	1.01±0.30	0.40±0.20	0.80±0.35
Ole4	1	0.72±0.22	0.53±0.26	0.75±0.24

TaqMan qPCR reaction mixtures (25 µl) contained 25 ng of RNA-equivalent cDNA from various stages of tung seeds, the optimized concentrations of each primer and probe (200 nM) and QPCR Mix. The expression levels under each tree represent the means and standard deviations of the expression fold calculated using three reference mRNA (Rpl19b, Gapdh and Ubl) from 11 stages of seeds with 2–4 assays for each stage. Ole gene expression in tree 1 seeds was used as the calibrator for the calculation of Ole gene expression in tree 2 and tree 3 seeds.

### OLE Gene Expression among Tung Tissues

TaqMan and SYBR Green qPCR was used to analyze OLE gene expression among tung tissues using cDNA from tung seeds, leaves and flowers with optimized primer pair and probe concentrations. Seeds had the highest expression levels for all five OLE genes by either qPCR assay (Table 4 and data not shown). TaqMan qPCR shows that OLE1, OLE2 and OLE3 mRNA levels were hundreds of folds higher in the seeds than those in the leaves and flowers, respectively, whereas OLE4 mRNA levels in the seeds were tens of folds higher than those in the leaves and several-fold higher than those in the flowers (Table 4). The mean C_T_ values of the TaqMan qPCR amplification were included in the table to illustrate the existence of OLE mRNA in the leaves and flowers. OLE5 gene expression was similar to that of OLE4 in tung tissues (data not shown). SYBR Green qPCR, which utilizes different detection chemistry, was used to confirm the results from TaqMan qPCR assays. SYBR Green qPCR assays generated similar expression patterns in the three tissues (Table 4). Although the expression levels of OLE genes in tung leaves and flowers were low, melt curve analysis and agarose gel electrophoresis showed that these SYBR Green qPCR generated DNA amplicons matched the predicted sizes (Figure 3B and Figure S3 vs. Table 1).

**Table 4:** Ole gene expression among tung tissues.

qPCR method	mRNA	Seed	Leaf	Flower
		fold	fold (C_T_)	fold (C_T_)
TaqMan	Ole1	1	0.0002±0.0001 (33.58)	0.00003±0.00001 (36.18)
	Ole2	1	0.0006±0.0004 (32.52)	0.0003±0.0001 (33.43)
	Ole3	1	0.0007±0.0004 (32.28)	0.0056±0.0023 (28.87)
	Ole4	1	0.0206±0.0116 (32.62)	0.1247±0.0514 (29.67)
SYBR Green	Ole1	1	0.0004±0.0002 (28.14)	0.0002±0.0001 (29.74)
	Ole2	1	0.0010±0.0005 (27.34)	0.0005±0.0002 (28.55)
	Ole3	1	0.0007±0.0003 (28.36)	0.0061±0.0031 (25.39)
	Ole4	1	0.0473±0.0221 (25.84)	0.4323±0.2201 (22.73)

The qPCR reaction mixtures contained 5 ng of RNA-equivalent cDNA from various stages of tung tree 1 seeds, leaves and flowers, the optimized concentrations of each primer and probe (200 nM) and QPCR Mix. The expression levels under “seed” represent the means of the expression fold of 4 stages of seeds (weeks 2, 4, 6 and 10) (TaqMan qPCR) or 6 stages of seeds (weeks 2, 4, 5, 6, 8 and 10) (SYBR Green qPCR) calculated using three reference mRNA (Rpl19b, Gapdh and Ubl) with 2–4 assays for each stage. The expression levels under “leaf” and “flower” represent the means and standard deviations of the expression fold calculated using three reference mRNA (Rpl19b, Gapdh and Ubl) each with 2–4 assays and Ole gene expression in seeds as the calibrator. The mean C_T_ values generated using RNA from leaves and flowers are included after expression fold as a proof of low but reliable levels of Ole mRNA detection in these tissues.

### TaqMan qPCR Analysis of OLE Gene Expression in Developing Tung Seeds

**Figure 4:**
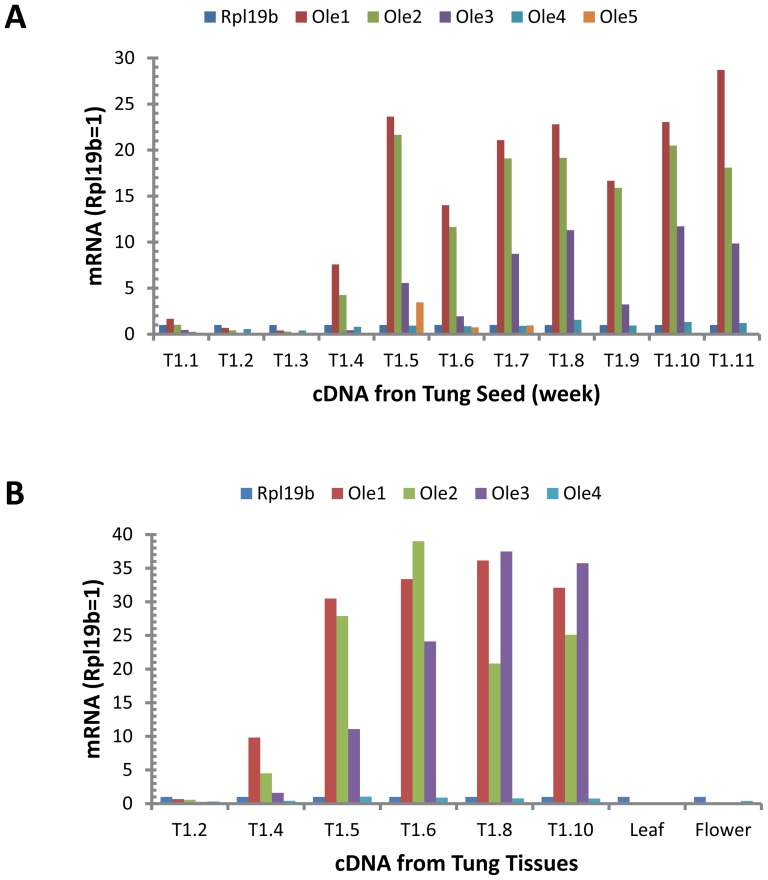
Relative levels of OLE gene expression in developing tung seeds, leaves and flowers. (A) TaqMan qPCR. The qPCR reaction mixtures contained 25 ng of RNA-equivalent cDNA from tung seeds and 200 nM of each primer and probe. (B) SYBR Green qPCR. The qPCR reaction mixtures contained 5 ng of RNA-equivalent cDNA from various stages of tung seed, leaves and flowers and 200 nM of each primer. The means of mRNA expression levels calculated from two qPCR assays in each seed stage using Rpl19b as the reference mRNA is presented. The results using Gapdh and Ubl as the reference mRNA are presented in Figure S5 (TaqMan qPCR assay) and Figure S6 (SYBR Green qPCR assay).

TaqMan qPCR assays were performed under optimal concentrations of primers and probe for evaluating the expression profiles of the five OLE genes in developing tung seeds using reference genes Rpl19b, Gapdh and Ubl. Figure 4A shows that the expression of OLE1 and OLE2 genes dramatically increased following the initiation of seed development, which were detectable at week 4 seeds and increased significantly at week 5 seeds. OLE3 mRNA levels were detected in week 5 seeds with lower levels than those of OLE1 and OLE2 mRNA (Figure 4A). While OLE4 mRNA levels were relatively low, OLE5 gene expression in tung seeds was minimal (Figure 4A). Similar profiles of OLE gene expression in tung seeds were observed using the three reference genes Rpl19b (Figure 4A), Gapdh (Figure S5A) and Ubl (Figure S5B).

Among the individual OLE genes, OLE1 mRNA levels were increased 11-, 40- and 97-fold in stage 4, 6 and 10 seeds compared to those in stage 2 seeds (Table S3). Similar increases of OLE2 mRNA levels were observed in the seeds. OLE3 mRNA levels were upregulated even more dramatically, with 7-, 41- and 413-fold increases in stage 4, 6 and 10 seeds compared to stage 2 seeds (Table S3). In contrast, OLE4 mRNA levels were only increased 1.2-, 3.1- and 6.7-fold in stage 4, 6 and 10 seeds compared to stage 2 seeds (Table S3). Interestingly, OLE5 mRNA levels were increased 16- and 18-fold in stage 4 and 6 seeds but declined to 0.23-fold in stage 10 seeds compared to stage 2 seeds (Table S3).

### SYBR Green qPCR Analysis of OLE Gene Expression in Tung Tissues

OLE gene expression in different stages of seed development was also evaluated by the optimized and verified SYBR Green qPCR assay using the reference genes Rpl19b, Gapdh and Ubl. Figure 4B shows that the expression of OLE1, OLE2 and OLE3 genes was detectable at week 4 seeds and increased significantly at week 5 seeds. OLE4 mRNA levels were relatively low in tung seeds (Figure 4B). OLE mRNA levels were detectable in tung leaves and flowers at extremely low levels compared to those in tung seeds (Figure 4B vs. Table 4). Similar profiles of OLE gene expression in tung tissues were observed using the three reference genes Rpl19b (Figure 4B), Gapdh (Figure S6A) and Ubl (Figure S6B). SYBR Green qPCR results were in general agreement with TaqMan qPCR except that SYBR Green qPCR detected higher relative levels of OLE3 mRNA in the seeds than TaqMan qPCR. These differences in relative quantification of OLE mRNA might be due to the different chemistries of the two qPCR assays utilized [49], [52].

OLE1 mRNA levels were increased approximately 15-, 80- and 108-fold, whereas OLE3 mRNA levels were increased by 8-, 110- and 99-fold in stage 4, 6 and 10 seeds compared to stage 2 seeds (Table S3). OLE3 mRNA levels were increased more dramatically with 6-, 148- and 306-fold in stage 4, 6 and 10 seeds compared to stage 2 seeds (Table S3). In contrast, OLE4 mRNA levels were only slightly increased to 1.4-, 4.6- and 5.6-fold in stage 4, 6 and 10 seeds compared to stage 2 seeds (Table S3).

## Discussion

Tung tree is a tropical plant with a very limited growing area in the United States [1], [2]. Tung orchards in the southern United States were largely destroyed by hurricanes including Hurricanes Betsy in 1965, Camille in 1969, and Katrina and Rita in 2005. The losses of tung orchards due to hurricanes spurred interest in trying to preserve a reliable domestic source of tung oil in the US by engineering tung oil biosynthetic pathway in traditional, temperate oilseeds. To this end, multiple isoforms of many gene families from the tung oil biosynthetic pathway have been cloned in our laboratories in recent years.

In this study, we identified five tung OLE genes by EST sequencing and pyrosequencing (“454”) technology using anonymous tung cDNA sequences from week 6 seeds. All five OLE shared significant conservation at the protein and nucleotide levels. The five OLE genes coded for small proteins with an average of 154 amino acid residues and an average of 16.5 kDa. They possessed high isoelectric point and high percentage of hydrophobic residues. These properties of OLE proteins were similar to those of the three DGAT in tung tree [13] and DGAT in other plants, animals and fungi [24], [80]. OLE1 and OLE2 were identical to those isolated from cDNA library of tung seeds (OleI-GU245884 and OleII-GU245885) and EST (vf3-GR217899 and vf2-GR217906) [35], [36]. OLE3 and OLE5 are identical to those isolated from cDNA library of tung seed EST (vf1-GR217754 and vf5-GR218198) [35]. The reported vf4 has an N-terminal extension and only six varied amino acid residues at the C-terminus of the protein compared to vf3 and clustered together in the phylogenetic tree [35], suggesting that these two genes constitute a paralogous pair, a common occurrence in eudicot species such as *Arabidopsis* [71]. It might be preferable to name the reported vf3 and vf4 as OLE3a and OLE3b, respectively. Therefore, we named the new OLE as OLE4 in this report since only five major groups of OLE were present in tree species.

The numbers of OLE genes in plants are widely different. We performed a genome-wide search for OLE genes in the sequenced tree genomes including *Prunus persica* (peach) [37], *Populus trichocarpa* (poplar) [38], *Ricinus communis* (castor bean) [39], *Theobroma cacao* (cacao) [40] and *Vitis vinifera* (grapevine) [41]. The completed genome sequences from the five trees provide a great opportunity for classifying tree OLE proteins. Phylogenetic analysis of all OLE clearly grouped these proteins into five subfamilies. Each of the five tung OLE genes grouped with each of the five OLE subfamilies. These results suggest that tung tree might only contain five subfamilies of OLE and this report might represent the complete OLE gene family in tung tree. Interestingly, the five tung OLE only aligned with the S and SM type but not the T type of *Arabidopsis* OLE. Tung OLE1, OLE2 and OLE3 belong to the S type and OLE4 and OLE5 belong to the SM type of *Arabidopsis* OLE. The more abundant expression of OLE1, OLE2 and OLE3 than OLE4 and OLE5 in tung tree seed support the classification of tung OLE based on *Arabidopsis* OLE.

It is important to determine which isoform is expressed in developing seeds to understand the genetic control of oil biosynthesis and to guide rational design of successful transgenic plants. In this report, we analyzed expression of OLE gene family quantitatively in 3 tung trees, 3 tung tissues (seeds, leaves and flowers) and 11 stages of seed development. Under optimized assay conditions, qPCR exhibited similar amplification efficiencies between the OLE genes (Figure 4C, Figure S4 and Table S1) and the three reference genes reported previously [51], which allowed for comparison of the relative expression levels in different tissues and developmental stages. This study provides complete expression profiles of the OLE genes in tung tissues. Both qPCR methods show that OLE genes were mainly expressed in developing tung seeds and that low levels of OLE mRNA were detected in tung leaves and flowers. OLE1, OLE2 and OLE3 were the major OLE mRNAs in the seeds whose mRNA levels were rapidly increased in developing seeds when tung oil begins to accumulate and their expression levels are sustained in all subsequent stages [13]. These developmental expression profiles of OLE genes in tung tree seeds are very similar to those in the seeds of *Coffea canephora* [55] and *Brassica napus* [81].

It is noteworthy that OLE transcripts were detected in leaves and flowers of tung tree, although their mRNA levels were extremely low compared to those in the seeds. The expression of OLE genes in these non-seed tissues was verified by melt curve and gel electrophoresis analyses of the qPCR products with predicted sizes of the amplicons. It was generally accepted that OLE genes was only expressed in the seeds, pollen and tapetum but not in other tissues [31], [82]–[84]. This was supported by the studies of OLE gene expression in *Arabidopsis* [72] and olive [65]. However, recent studies provided evidence for the expression of OLE1 and OLE2 but not OLE3 genes in all tissues of the moss *Physcomitrella patens* [85]. SYBR Green qPCR revealed weak expression of some forms of OLE genes in cotyledon and young leaves of flax but not castor bean [86]. TaqMan qPCR method also detected significant amount of OLE5 mRNA in the mature flowers of *Coffea arabica* and *Caffea canephora*; in addition, one OLE5 EST was found in the EST library of *Caffea* leaves [55]. Low levels of an OLE-like transcript were found in transcriptomes and by RT-PCR in vegetative cells of *C. reinhardtii* grown under acetate-enriched medium [87]. These expression data support the idea that oil body biogenesis is present in tissues other than seeds such as tobacco leaf [88] and olive fruit [89]. These results demonstrate that OLE genes are expressed in tissues other than the seeds, pollen and tapetum, and suggest that they may play a role in these tissues.

### Conclusions and Future Research

We identified five OLE genes in tung tree. Genome-wide phylogenetic analysis and multiple sequence alignment demonstrated that the five tung OLE represented the five OLE subfamilies and contained the “proline knot” motif (PX5SPX3P) shared among 65 OLE from 19 tree species. Tung OLE1, OLE2 and OLE3 belong to the S type and OLE4 and OLE5 belong to the SM type of *Arabidopsis* OLE. Expression results demonstrated that all five OLE genes were expressed in developing tung seeds, leaves and flowers but their expression levels were much higher in the seeds than leaves or flowers. In addition, OLE1, OLE2 and OLE3 genes were expressed in tung seeds at much higher levels than OLE4 and OLE5 genes. Finally, the amounts of OLE mRNAs were rapidly increased in developing seeds and their expression levels were well-coordinated with tung oil accumulation. The information on OLE expression profiles suggests that OLE1, OLE2 and OLE3 genes may play major roles in tung oil biosynthesis and/or tung oil body development. Therefore, they might be preferred targets for tung oil engineering in transgenic plants.

There is no information about the genes coding for other oil-body proteins including caleosins and steroleosins in tung tree at this time. Identification and characterization of additional oil body proteins and their corresponding genes at the genomics and proteomics levels would enhance the understanding of genetic and mechanistic control of tung oil biosynthesis. This information is essential for improving important oils such as tung oil in the future.

## Supporting Information

Figure S1 **Nucleotide sequence alignment of the five Ole genes in tung tree.** Multiple sequence alignment was performed using the ClustalW algorithm of the AlignX program of the Vector NTI software. Ole sequence name is on the left of alignment followed by the GenBank accession number and the start of the nucleotide sequence of each Ole gene. The numbers at the top of the alignment are the positions of the multiple sequence alignment. The letters at the bottom of the alignment are the consensus nucleotides. Nucleotides in red on yellow represent those conserved in all five Ole sequences at a given position, whereas those in black on blue represent nucleotides conserved in majority of the sequences at a given position. The underlined nucleotides represent the forward primers, TaqMan probes and the complementary sequences of the reverse primers used in qPCR assays.
(PDF)

Figure S2 **Multiple sequence alignment for the identification of amino acid residues and sequence motifs conserved in OLE.** Multiple sequence alignment was performed using the ClustalW algorithm of the AlignX program of the Vector NTI software. Each OLE sequence name is on the left of the alignment followed by the position of amino acid residue of OLE protein sequence in the alignment. The letters at the bottom of the alignment are the consensus residues. Color codes for amino acid residues are as follows: 1) red on yellow: consensus residue derived from a completely conserved residue at a given position; 2) black on green: consensus residue derived from the occurrence of greater than 50% of a single residue at a given position; 3) blue on cyan: consensus residue derived from a block of similar residues at a given position; 4) green on white: residue weakly similar to consensus residue at a given position; 5) black on white: non-similar residues. The abbreviations of the organisms are: Car, *Coffea arabica* (coffee); Cca, *Coffea canephora* (coffee); Cav, *Corylus avellana* (hazelnut); Col, *Camellia oleifera* (tea oil); *Citrus sinensis* (orange); Egu, *Elaeis guineensis* (oil palm); Fpu, *Ficus pumila* (climbing fig); Jcu, *Jatropha curcas* (barbados nut); Jre, *Juglans regia* (walnut); Oeu, *Olea europaea* (olive); Pam, *Persea Americana* (avocado); Pdu, *Prunus dulcis* (almond); Ppe, *Prunus persica* (peach); Pta, *Pinus taeda* (loblolly pine); Ptr, *Populus trichocarpa* (poplar); Rco, *Ricinus communis* (castor bean); Tca, *Theobroma cacao* (cacao); Vfo, *Vernicia fordii* (tung tree); Vvi, *Vitis vinifera* (grapevine).
(PDF)

Figure S3 **Specificity of SYBR Green qPCR Assay.** The qPCR reactions contained 5 ng RNA-equivalent cDNA from tung tree leaves and flowers. The qPCR products were separated by agarose gel electrophoresis. Lane 100 bp represents DNA ladders with 100 bp as the smallest band, increasing upward in 100 bp increments. The results using tung tree seeds are shown in Figure 3B. (A) Melt curve analysis, (B) Gel electrophoresis.
(PDF)

Figure S4 **qPCR efficiency for OLE assay.** TaqMan and SYBR Green qPCR reaction mixtures contained variable concentrations of RNA-equivalent cDNA from tung seeds, the optimized concentrations of each primer and probe (200 nM), and Absolute QPCR Mix (TaqMan qPCR) or each primer and 1 x iQ SYBR Green Supermix (SYBR Green qPCR). The results using RNA isolated from stage 4 seed of tree 1 are shown. The qPCR efficiency for Ole1 mRNA detection is presented in Figure 3C. The results using RNA from other stages of tung seeds, leaves and flowers are presented in Table S1. (A) qPCR efficiency for Ole1 mRNA detection, (B) qPCR efficiency for Ole2 mRNA detection, (C) qPCR efficiency for Ole3 mRNA detection, (D) qPCR efficiency for Ole4 mRNA detection.
(PDF)

Figure S5 **Relative levels of OLE gene expression in developing tung seeds by TaqMan qPCR.** The qPCR reaction mixtures contained 25 ng of RNA-equivalent cDNA from tung seeds and 200 nM of each primer and probe. The means of mRNA expression levels calculated from two qPCR assays in each seed stage using Rpl19b as the reference mRNA are presented in Figure 4A. The means of mRNA expression levels calculated from two qPCR assays in each seed stage are presented. (A) Gapdh as the reference mRNA, (B) Ubl as the reference mRNA.
(PDF)

Figure S6 **Relative levels of OLE gene expression in tung tissues by SYBR Green qPCR.** The qPCR reaction mixtures contained 5 ng of RNA-equivalent cDNA from various stages of tung seed, leaves and flowers and 200 nM of each primer. The means of mRNA expression levels calculated from two qPCR assays in each seed stage using Rpl19b as the reference mRNA are presented in Figure 4B. The means of mRNA expression levels calculated from two qPCR assays in each seed stage are presented. (A) Gapdh as the reference mRNA, (B) Ubl as the reference mRNA.
(PDF)

Table S1 **TaqMan qPCR efficiency for quantifying Ole mRNA in tung tree tissues.**
(PDF)

Table S2 **Variation of Ole gene expression among tung trees.**
(PDF)

Table S3 **Ole gene expression among different stages of tung seeds.**
(PDF)
