# Supplementary material for: Identification, Classification and Differential Expression of Oleosin Genes in Tung Tree (Vernicia fordii)
Source: PLoS One. 2014 Feb 6;9(2):e88409. doi: 10.1371/journal.pone.0088409 (PMC3916434; doi:10.1371/journal.pone.0088409)
Supplement: Figure S1 — Nucleotide sequence alignment of the five Ole genes in tung tree. Multiple sequence alignment was performed using the ClustalW algorithm of the AlignX program of the Vector NTI software. Ole sequence name is on the left of alignment followed by the GenBank accession number and the start of the nucleotide sequence of each Ole gene. The numbers at the top of the alignment are the positions of the multiple sequence alignment. The letters at the bottom of the alignment are the consensus nucleotides. Nucleotides in red on yellow represent those conserved in all five Ole sequences at a given position, whereas those in black on blue represent nucleotides conserved in majority of the sequences at a given position. The underlined nucleotides represent the forward primers, TaqMan probes and the complementary sequences of the reverse primers used in qPCR assays. (PDF) [file pone.0088409.s001.pdf]

```
                                 1                                                                                                  100
VfOle1 (GU245844.1)       (1)    ---------------------------------------------------------------------------------------------------A
VfOle2 (GU245885.1)       (1)    --------------------------------------------------------------------------------------------ATTCCACA
VfOle3 (GR218754.1)       (1)    -----------------------GCTCTTTCTCATTTTCTCTGCAAAAATCTAATCTTTTTCTATACTTTCCAA---CAAAAGCAGTACAATTAAGCCTA
       VfOle4 (new)       (1)    -------------------------------------TGGTCCTATTTACGGCCGGGGGAACCTTAAACTCAATTTTGTCTCCCATGGCTGATCGTACTC
VfOle5 (GR218198.1)       (1)    TCGTGCCGAATTCGGCACGAGGCTTTTCTTATAATTGCAGTACGCATTACGAGAAGATCAAATTTATATTTCAATCCCAAATCCTCTCTTCTCTATCCTT
          Consensus       (1)                                            T C     A               TA   T  A          C            CCTA
                                 101                                                                                                200
VfOle1 (GU245844.1)       (2)    TTCAT----CAATTAATCATAAGGCCAAGGCATCAATCA--ATGGCAGAACAGCAACAG------TC-----ACAGCA-ACCTGTCCAACA--ACCACGC
VfOle2 (GU245885.1)       (9)    TTTATCATCAATGGCTGAACGTTTACAGCCACACCAGGTTCAAGTCCACCCACAACTCCGCTATGAT---GCCTACAAGGGTCAACAAAAGCGTCCATCA
VfOle3 (GR218754.1)      (76)    GCTAGC-TCCATGGCCGACTATCAGCAACGACCTGGCCAAGAAGGCTACAAAGGCGGCGTCTGGTCCCGTGATCAAAAGGGTCCTGGATCTGGCCCTTCA
       VfOle4 (new)      (64)    CTTCTCAGAGACTTTCAAGACTCTCCACCGTCGCCGCTACAACCACCGCCAATGGTTCTACTTTTCT-----ACGTAAACTTCAAGCTCACGCGCCTGAC
VfOle5 (GR218198.1)     (101)    TTTATA-CCCACACACTCTCCATTCCAACCAAATTATCATCATGTCTGATCAATCAAAGCCTATGTC-----TCAGATGATCCGTGAAGCAGCACCACCG
          Consensus     (101)    TTTATC  CCA    C A   T TCCAACGAC C A CA  A G C GC CA     CG CT T  C       CA AAG  TC TG A C G  CCA C
                                 201                                                                                                300
VfOle1 (GU245844.1)      (82)    TCTCACCAGGTAGTCAAGGCAGCAACTGCCGTCACTGCTGGTGGTTCCCTTCTAGTCCTCTCTGGTTTAACTCTCACTGGGACTGTCATTGGACTCACTA
VfOle2 (GU245885.1)     (106)    ACCTCTAAGGTGTTAGCTGTGATCACCCTTCTCCCCCTTGGTGGTGGGCTTCTTGCACTTTCAGGTATAACCCTAGTAGGTACACTCATCGGCCTAGCCA
VfOle3 (GR218754.1)     (175)    ACTTCACAGATTTTAGCTGTTGTCACTCTGTTGCCTGTTAGTGGGACCCTACTCTTCCTTGCTGGTATCACTCTTACTGGGACCCTCATTGGGTTAGCTG
       VfOle4 (new)     (159)    TCAACCCAACTCCTTGGTTTCCTGACTCTTTTAATTACTGGCTCTATTCTTCTCCTTCTTGCAGGCATCACCGTCACTGTTGTAGTTTTTTCTATAATCT
VfOle5 (GR218198.1)     (195)    TCTCACCCGACGGTCAAGTTCTTAACAGCAGCCACGCTTGGTGCAGCTTTCTTGATCTTGTCTGGGTTAACACTAACTGGGACAGTAATAGCTCTTATTA
          Consensus     (201)    TCT CCCAG T  T G TGT  T ACTCT  TCACT TTGGTGGT C CTTCT  TCCTTTCTGGTATAAC CT ACTGGGACAGTCATTGG CTAACTA
                                 301                                                                                                400
VfOle1 (GU245844.1)     (182)    TAGCCACTCCATTGTTCGTCCTTTTCAGTCCGGTTCTTGTTCCTGCTGTGATTACAGTTTTTCTTATAGTCACAGGCTTCTTGGCCTCTGGTGGTTTTGG
VfOle2 (GU245885.1)     (206)    TTACCACCCCTCTTTTTTTCATCTTTAGCCCTGTTCTTGTTCCGGCAGCCTTTGTTCTTGGCCTTGCTGTGATGACATTTTTGGCTTCTGGGGCTATGGG
VfOle3 (GR218754.1)     (275)    TGACTACCCCACTTTTTGTTATTTTCAGCCCCGTTCTGGTTCCGGCAGCCTTCGTTATTGGCTTGTCGGTGATGGGTTTTTTGGCCTCTGGAGCTTTTGG
       VfOle4 (new)     (259)    TTTTTACTCCTCTGATTATAATTTCTAGTCCCATATGGTTCCCAATCGCCGTCATCTTATTTATCTCCGTCGCTGGATTTTTATCCATTTGTGGATTCGT
VfOle5 (GR218198.1)     (295)    TGGCAACTCCAATTCTGGTCCTGTTTAGTCCTATTATAGTCCCAGCTGGGATAGTTGTATTCTTGGTAGCTACTGGGTTCTTTTTTTCCGGTGGGTGTGG
          Consensus     (301)    T  C ACTCCACTTTTTGTCATTTTTAGTCC GTTCT GTTCC GC GCC T GTT TTTTC T  C GT AC GG TTTTTGGCCTCTGGTGGTTTTGG
                                 401                                                                                                500
VfOle1 (GU245844.1)     (282)    CGTCGCAGCAATCTCCGTCCTCTCATGGATCTACAGGTATGTGACCGGGAAGC-AACCGCCAGGAGCAGAAA-GTCTGGACCAGGCACGCATGAAGCTGG
VfOle2 (GU245885.1)     (306)    GCTAACAGGGCTGACTTCGCTTTCATGGGTTTTGAAGTACGTCC--AGGAGGCCACTCGGAACATACCTGAGCAGCTGGATCAGGCAAGGAGGCGCATGC
VfOle3 (GR218754.1)     (375)    GATCAGTGCACTTTCTTCTCTGTCTTGGATGGTTAATTATATCCGTAGGATG--AGAGGCTCTTTGCCGCTGCAGATGGAACATGCTAAGCGGCGCGTGC
       VfOle4 (new)     (359)    TGCGGCGGTTGTGGGTGGATTATCTTGGGCTTATAGGTATTTTAGAGGTATGA-ATCCACCCGGTTCAGACC-AGTTCGATTATGCTCGGAGTCGAATTT
VfOle5 (GR218198.1)     (395)    TTTGGCGGCGATAATGGCATTAACTTGGATGTATAATTACTTGACTGGGAAAC-ACCCGCCTGGTGCTGATA-AGTTGGATTATGCGAGAGGCAAAATTG
          Consensus     (401)      T GC GC  T  CTGC CT TCTTGGAT TATAAGTAT T A  GGGA GC A CCGCC GGTGC GA   AG TGGATCATGC AGGAGGCG ATG
                                 501                                                                                                600
VfOle1 (GU245844.1)     (380)    CTGGAAAGGCACGGGAAATGAAAGACAGGGCTGAGCAGTTAGGACAGCATGTAACGGGCCAACATACTTGAGAAGTGCTTTTGATGGAATA-TATATA-A
VfOle2 (GU245885.1)     (404)    AAGATATGGCTGGTTATGTGGGACAGAAGACTAAGGAAATGGGACAAGAAATCCAGAAGAAG---ACACATGAAGGGAAATGAATCAAATGATAAGAG-G
VfOle3 (GR218754.1)     (473)    AGGACACTACTGGTCAATTGGGCCAGAAGGCTAGGGATATGGGCCAGACTGTCCAGAGTAAG---GCCCAAGAAGGTGGCAAGACTTAAT--TATTGT-T
       VfOle4 (new)     (457)    ATGACACGGCTAGTCATGTGAAGGACTATGCTAGGGAATATGGTGGGTATTTGCAGAGCAAGGTGAAGGATGCT--GCTCCTGGTGCATGATTATCGGAT
VfOle5 (GR218198.1)     (493)    CGAAAAAGGCACAGGATATGAAAGAATATGTCCAGCAAAAAGCTCAGGAGGCTACTACTCAA---ACTCGAGCA-TCTTAATTATTAATTA-TATATG-T
          Consensus     (501)    A GA A GGCT GT AT TGAAAGA AAGGCTAAGGAAAT GG CAG ATGT CAGAG AAG   AC CAAGAAG G T  TGAT  AATA TAT  G T
                                 601                                                                                                700
VfOle1 (GU245844.1)     (478)    AATGGCTTTGATAT------CTCTTTGTCT-TTAATTTAAATTTGG---AAGGCCATTGCTTTGCT-----TTTG-TCTCCAATGTTTCGTTTTTTTGGG
VfOle2 (GU245885.1)     (500)    AATGTTGTTGGTTTATAAATGTTTTTGTTTTCAAATAGAGAGCTGTGAGGGGGTACTTTTGTAGGAGGGGTCTTG-TTGTAAGGTAGATGAGTTGGTTCA
VfOle3 (GR218754.1)     (567)    AATGGTTTTGGT--------TTTGGAGTTTGATAAAGGATCTCACTCATAAAATTATGTAATAAGA-----CTTG-TTTGTGT--A----------T---
       VfOle4 (new)     (555)    CATGGATATCGGATCG---GGTTTTAATCA---AGTTTATATATATTTTAATTTTATTTTTGGGTTTTTGATCTGATTATTATCCATTTTGAATGATGGA
VfOle5 (GR218198.1)     (587)    ATTTGTTTTGCTAA------TTAGTAATTAGTTAGTACAGTTTTGGCAAAAAAAAAAATAGTTAGT-----ACTG-TTTTTGTTAATTTCAACTCTTTTT
          Consensus     (601)    AATGGTTTTGGTAT       TTTTAGTTT  TAAT  A AT TGT A AA  T ATTT  T GGT      TTG TTTTTAT  ATTT    T  T
                                 701                                                                                                800
VfOle1 (GU245844.1)     (562)    TTAATCTTTTTTTTTTTGTTTTTTTTAATGG--GATCTTT-TGTGAGTTTTT-TCT-----TTTTAAAT-----TAAATAATTTCTTTGTCTTTGTGTTA
VfOle2 (GU245885.1)     (599)    TGGGCTCTCCTTTGTTGCATTTGTGTCGTTT-TATTTTGTCTTTTGGGTTCAATTT-----TTTATTTTCAATGTATTAATCTTGTCTGTTTCATCATTC
VfOle3 (GR218754.1)     (638)    --AGCCCTTTTTTTTTTCTTTTTTTTCATAAATGCTATGT--TTGGGTTTCTGTGT-----TTGTTAATC---AAATAAATTTTATTTAAGTTTATTCTC
       VfOle4 (new)     (649)    ATA--TTTATATTTAATGTTTGTTTTAATGGATATTAAAATGGTTAATGTTTGTATGGAAGTTTTGATGCCTTATTTGAATATTCTTCTTCGTTGGATTA
VfOle5 (GR218198.1)     (675)    TTA-TTTTTATTTTTTATTTTTTTATGTTGATTGATGTTGATGTGGGGTTGT-TGT-----TTTTGAAGCTG-ATGAAAATGATCTTTCCATCTATGTTA
          Consensus     (701)    TTA  TTTTTTTTTTTT TTTTTTTT ATG  TG T T T TGTGGGTTT T T T     TTTT AATC   ATATAAAT TTCTTT T TTT T TTA
                                 801                                                                                                900
VfOle1 (GU245844.1)     (648)    AACTTAATCTTTACTCGTAATCAAGTCTTATTTTATTTCATTGCTGTTTAATTAATTAATTAGTTTGTTAAAAAAAAAAAAAAAAAAAAAA---------
VfOle2 (GU245885.1)     (693)    TCCCTCTCTCACACCCACACCCACCATGATTCGTTTCTTATATCAGGTTCAGGAGTCTATTGTTCAGTTTATCCTTGCAGTTCAGTAAAATAAATAAAGA
VfOle3 (GR218754.1)     (726)    T---------------------------------------------------------------------------------------------------
       VfOle4 (new)     (747)    G--GAACATGATAGAAATCCTAAATTTTGTACTAATTATCATTTGTTTTTCAATTAGTGTGCTTTTCTTCTTCT--------------------------
VfOle5 (GR218198.1)     (767)    A--TAAATCTTT--TAATAATCAAAT--TGAGTTATTTGACT----------------------------------------------------------
          Consensus     (801)        A      TA   ATA TCAA T  T T  TATTT A     G TT    A T   T        T
                                 901                                         946
VfOle1 (GU245844.1)     (739)    ----------------------------------------------
VfOle2 (GU245885.1)     (793)    AAGAAAAGCACTGATCCTAGTTTGCCACTAAAAAAAAAAAAAAAAA
VfOle3 (GR218754.1)     (727)    ----------------------------------------------
       VfOle4 (new)     (821)    ----------------------------------------------
VfOle5 (GR218198.1)     (803)    ----------------------------------------------
          Consensus     (901)
```

Figure S1. Nucleotide sequence alignment of the five Ole genes in tung tree. Multiple sequence alignment was performed using the ClustalW algorithm of the AlignX program of the Vector NTI software. Ole sequence name is on the left of alignment followed by the GenBank accession number and the start of the nucleotide sequence of each Ole gene. The numbers at the top of the alignment are the positions of the multiple sequence alignment. The letters at the bottom of the alignment are the consensus nucleotides. Nucleotides in red on yellow represent those conserved in all five Ole sequences at a given position, whereas those in black on blue represent nucleotides conserved in majority of the sequences at a given position. The underlined nucleotides represent the forward primers, TaqMan probes and the complementary sequences of the reverse primers used in qPCR assays.
