# Supplementary material for: Identification, Classification and Differential Expression of Oleosin Genes in Tung Tree (Vernicia fordii)
Source: PLoS One. 2014 Feb 6;9(2):e88409. doi: 10.1371/journal.pone.0088409 (PMC3916434; doi:10.1371/journal.pone.0088409)
Supplement: Figure S2 — Multiple sequence alignment for the identification of amino acid residues and sequence motifs conserved in OLE. Multiple sequence alignment was performed using the ClustalW algorithm of the AlignX program of the Vector NTI software. Each OLE sequence name is on the left of the alignment followed by the position of amino acid residue of OLE protein sequence in the alignment. The letters at the bottom of the alignment are the consensus residues. Color codes for amino acid residues are as follows: 1) red on yellow: consensus residue derived from a completely conserved residue at a given position; 2) black on green: consensus residue derived from the occurrence of greater than 50% of a single residue at a given position; 3) blue on cyan: consensus residue derived from a block of similar residues at a given position; 4) green on white: residue weakly similar to consensus residue at a given position; 5) black on white: non-similar residues. The abbreviations of the organisms are: Car, Coffea arabica (coffee); Cca, Coffea canephora (coffee); Cav, Corylus avellana (hazelnut); Col, Camellia oleifera (tea oil); Citrus sinensis (orange); Egu, Elaeis guineensis (oil palm); Fpu, Ficus pumila (climbing fig); Jcu, Jatropha curcas (barbados nut); Jre, Juglans regia (walnut); Oeu, Olea europaea (olive); Pam, Persea Americana (avocado); Pdu, Prunus dulcis (almond); Ppe, Prunus persica (peach); Pta, Pinus taeda (loblolly pine); Ptr, Populus trichocarpa (poplar); Rco, Ricinus communis (castor bean); Tca, Theobroma cacao (cacao); Vfo, Vernicia fordii (tung tree); Vvi, Vitis vinifera (grapevine). (PDF) [file pone.0088409.s002.pdf]

```
                           1                                                                                                  100
CarOle-AAY14574       (1)  ----------------MAEHYQLQQRPTEAV--K----SFLPQKGP----STSHVLAVVTLLPVAGVLLGLSGLILVGTVIGLAVTTPLFVIFSPILVPA
CcaOle-AAX49389       (1)  ----------------MAEHYQLQQRPTEAV--K----SFLPQKGP----STSHVLAVVTLLPVAGVLLGLSGLILVGTVIGLAVTTPLFVIFSPILVPA
ColOle-ABF57559       (1)  --------------MAEQRIQDYQQKATDNF--K----GFLPEKGP----SKSQILAVVTLLPVGGFLLLLSGLTLTGTLIGLAVTTPLFVICSPVLVPA
VviOle-XP_002281381   (1)  ----------------MAEHQQRP--------------GFMPEKGP----STSQVLAVITLFPVGGILLVLSGLTLAGTLIGLTVATPLFVIFSPILVPA
JcuOle-ABW90148       (1)  ----------------MAEYERRPGGEGHKGALK--------EKGG---FSTSQILAVVTLLPVSGTLLFLAGITLTGTLIALAVATPLFVICSPVRGPA
RcoOle-XP_002511342   (1)  -------------MAERLQQQQQQG---------------HDKKGP----STSQILAVVTLLPLSGTLLFLAGATLVGALTGLGVTTPLFVIFSPVLVPA
VfoOle3-GR217899.1    (1)  --------------METADYQQRPGQEGYKGGVWS-----RDQKGPGSGPSTSQILAVVTLLPVSGTLLFLAGITLTGTLIGLAVTTPLFVIFSPVLVPA
PtrOle-XP_002318659   (1)  -----------------QQHQQRPGGQGFASTLP--------GKGP----STSQVLAVVTLLSVGGTLLFLSGLTLVGTLIGLAVTTPVFVIFSPVLVPT
PtrOlep-XP_002322186  (1)  --------------------------------------------------------------------------TLVGTLIGLAVATPLFVIFSPALVPA
PpeOle-EMJ10773       (1)  MAEQHPRPQEHQGYQTQHQYDQQQQHQGFQYDDQQQPKGFLPQNGP----SATHIVAMLTLVPIGGTLLFLSGLTLAGTILGLAVSTPLFVIFSPILVPA
CavOle-AAO65960       (1)  ----------------------MAEHP-------------RQLQDPAHQPRSHQVVKAATAATAGGSLLVPSGLILAGTVIALTLATPLFVIFSPVLVPA
JreOle-AET74076       (1)  ----------------------MAEHQ-------------QQSQHPAQQPRSHQVVKAATAATAGGSLLLLSGLILASTVIALTVVTPLLVIFSPVLVPA
JreOle-AET74077       (1)  ----------------------MAEHQ-------------QQSQRPDLQPRSHQVVKAATAATAGGSLLVLSGLILTGTVISLTVATPLFVIFSPVLVPA
ColOle-ABF57560       (1)  ----------------------MAEIY-------------PQQQQPTTEPRSHQVAKAATAATACGSLLILSALTLAGTVVALTLATPLLVIFSPVLVPA
VviOle-XP_002281746   (1)  -----------------MAEIQPPHHI-------------LHPHQPQPQQPSYQAVKAATAATAGGSLLVLSGLTLVGTVIALTVATPLLVIFSPVLVPA
CcaOle-AAX49390       (1)  ----------------------------------------MADIRQQ-QPLSHQVVKAATAVTAGGSLLVLSGLILAGTVIALALATPLLVIFSPVLVPA
ColOle-ABF57563       (1)  ----------------------MAEQF-------------QQRYQQQNQPQAHYMVKAATAITAGGSLLVLSGLTLAGTVIALALATPLLVIFSPVLVPA
FpuOle-ABQ57397       (1)  -----------MAEPQSLQRGERGEQL-------------QLQLQQQQHPRSHQVVKAATAVTAGGSLLVLSALILAGTVIALTIATPLFVIFSPVLVPA
PduOle-Q43804.1       (1)  ---------------MADQHFQQPLHF-------------QGSYGQQ-QPRSYQVAKAATAVTAGGSLLVLSGLVLAGTVIALTIATPLLVIFSPVLVPA
PpeOle-EMJ19764       (1)  ---------------MADQHFQQPLHF-------------QGSYGQQ-QPRSYQVAKAATAVTAGGSLLVLSGLVLAGTVIALTIATPLLVIFSPVLVPA
CsiOle-CAA88360       (1)  -------------------MAEHYQPL-------------QQTQLQSRQPRSHQVVKAATAVTAGGSLLVLSGLTMAGTVIALTIATPLLVICSPVLVPA
PtrOle-XP_002297927   (1)  -----------------------MAEL-------------QQSQQPGKQPRSQQIVKATTAVTAGGSLLVLSGLTLAATVILLTIATPLLVIFSPVIVPA
PtrOle-XP_002304591   (1)  -----------------------MADL-------------QKSKHPRQQPRSHQVVKATTAVTTGGSLLVVSSLTFTATVILLTVATPLLIIFSPVIVPA
JcuOle-ABW90150       (1)  -----------------------MAEH-------------PQSQHVGQQPRSHQVVKAATAVTAGGSLLVLSGLTLAGTVIALTVPTPLLVIFSPVMVPT
JcuOle-AFP19885       (1)  -----------------------MAEH-------------PQSQHVGQQPRSHQVVKAATAVTAGGSLLVLSGLTLAGTVIALTVATPLLVIFSPVLVPA
VfoOle1-GU245884      (1)  -----------------------MAEQ-------------QQSQQPVQQPRSHQVVKAATAVTAGGSLLVLSGLTLTGTVIGLTIATPLFVLFSPVLVPA
RcoOle-XP_002511014   (1)  -----------------------MAEH-------------QQSPVVSHRPRVNQLVKAGTAATAGSSLLFLSGLTLTGTVIALALATPLMVLFSPVLLPA
TcaOle-EOY22772       (1)  -------------------MAEHLQLQ-------------QQYHHPYSQPRSHQMVKAATAATAGGSLLILSGLILAGTVIALTIATPLFVIFSPVLVPA
EguOlep-AF273023_1    (1)  ------------------HEVIKSEEG-------------EVEQMAEQQPTSQRVVKGVAAATIGGSLLLVSGLTLAGTVIGLAVVTPLLVIFSPVLVPA
CcaOle-AAX49392       (1)  -------------------------MA-------------TLPDQPQTQHSTSQVVKTTTAVAVGGSLMLLSGLTLAGTIIGLVLATPLLVIFSPVIVPA
VviOle-XP_003631370   (1)  --------------------------M-------------AHQDQPQKTQLSYQLIKTSTAATIGGSCMVLSGLTLAGTVIALVVATPLLVIFSPVLVPA
PamOle-AGT63296       (1)  ----------------------MADQP-------------KTIKQTERAPNTNQTAIFLTAAAVGGTLLVLSGLTLTATAISLLMATPLLVIWSPILVPT
PpeOle-EMJ25876       (1)  --------------MADQSRHVTLKLQ-------------DHDQSAAPPSSLRLTGKFLTAGAAGTTLLVLSGLTLTGTVMALIMATPVLVVFSPILVPA
PpeOle-EMJ26259       (1)  ----------------------------------------NLYGRSAPPS--HQTVKLLTAAIVGATLLFLSGLTLTGTVIALIMATPVLVLFSPILVPA
PtrOle-XP_002309527   (1)  ------------------MPDRSRPMS-------------RYEPSSGQPTSRKAVK-FMTAGTIGAALLVLSGLTLTGTVIALVVATPVLVLSSPILVPA
PtrOle-XP_002334246   (1)  -------------------------MS-------------RYEQSSAQPTSRKAVLKFMTAGTIGAALLVLSGLTLTGTVIALIVATPILVLCSPVLVPA
VfoOle5-GR218198      (1)  --------------------MSDQSKP-------------MSQMIREAAPPSHPTVKFLTAATLGAAFLILSGLTLTGTVIALIMATPILVLFSPIIVPA
TcaOle-EOY29822       (1)  -------------------MSNDQNKP-------------MTQKLYESAPSSRQAAKFLTATTLGATLLFLSGLTLTGTVMALIMATPLMVIFSPILVPA
VviOle-XP_002275496   (1)  --------------------MSDQPKP-------------VTPKLYDSAPSSRQAVKFLTAATIGTILLVLSGLTLTGTVIALIIATPALVVFSPILVPA
CcaOle-AAX49393       (1)  ----MADHQASP-----GQPQWPPRPTTTSTAGAT--TLFLRKMHEHAP-NSTQLIGFLTLVISGGILLLLTGLTLTATVLGLIFFAPLIIISSPIWVPA
PpeOle-EMJ10882       (1)  ----MADRHQNQTQTQQQQPQRAPRPTNTNNPASTNASTFLRRLQGHAP-NSTQLVGFLTLLVSGAILLLLTGLTVTATILGLILVTPLIIISSPIWVPV
TcaOle-EOY20922       (1)  ----MAERNP-----SQQ--RAAPRP----NPTASAASTFLQKFQAHAP-NSAQLLGFLTLFVSASILITLTGLTVTAAILGLVIFMPLIVISSPIWIPV
PtrOle-XP_002317928   (1)  ----MADRAP---------TQRATRPTTTPTTHN--GSTFLRKLQVHIGSNSSQLVGLLTLIISGSILLLLTVITVTGSVLGLTLLTPLIIISSPIWIPI
RcoOle-XP_002511984   (1)  --MADRDRASNP-------AQRPTRTATSNATPNNHVSTFLRKLQSHAP-NSTQFMGILTLLISGSILLLLIGITVTALVMGLVFFTPLIIVSSPIWFPI
VfoOle4               (1)  --METADRTP---------SQRLSRLSTVAATTTANGSTFLRKLQAHAP-DSTQLLGFLTLLITGSILLLLAGITVTVVVFSIIFFTPLIIISSPIWFPI
VviOle-CBI15408       (1)  ----MADRHSGS-----GQTQRPPRPTPG--------SAFLRRLHDHAP-NSTQFIGFLALIVSGCILLFLTGLTITATVIGLIFFTPLILLSSPIWVPA
VviOle-XP_002273242   (1)  ----MADRHSGS-----GQTQRPPRPTPG--------SAFLRRLHDHAP-NSTQFIGFLALIVSGCILLFLTGLTITATVIGLIFFTPLILLSSPIWVPA
PtaOle-AFG55850       (1)  -----------------------------------------------------------------------------GTVIGLVVLTPVLIFFSPILIPV
PtaOle-AFG55851       (1)  -----------------------------------------------------------------------------GTVIGLVVLTPVLIFFSPILIPV
TcaOle-EOY20852       (1)  ----------------MAEYQQQNLQYPQQQPQTDNYNNYFQDNHP----STSKVLAVVTLLPVAGTLLGLAGLSLVGTLIGLAFAIPLFLLFSPVLVPA
VviOle-XP_002273158   (1)  -----------MAERDE-------------------------QKGP----SASKILAVIALLPLGGTLLLLSGLTFVGSMVGLAVATPLFLLFSPVLVPA
VviOle-XP_002275087   (1)  ----------------------------MADSPPTYEVEIQSQYHERGGLSISQILALITLLPTGGTLLLFAGLTFAASMVGLAVATPLFLIFSPVLVPA
VviOle-XP_002275021   (1)  -----------MADRPQP--HQLQVHPQHLYNAG--IKTLLPQKGP----STSQVLAVIALLPLAGFLLLLAGLTFAGSALGLAVITPLFLIFSPVLVPA
OeuOle-AAL92479       (1)  ---------MAERDRPQ--PHQVQVHTQSRYDQGGGMKSVLPKKGP----STSQVLAVVTLLPVGGTLLALAGLTLVESLIGLAVTTPLFIIFSPVLVPA
CavOle-AAO67349       (1)  -----------MADRPQ--QLQVHPQRGHGHYEG--GIKNQRGGGP----SAVKVMAVLAALPVGGTLLALAGLTLAGSVIGLLVTSPLFIIFSPVLVPA
TcaOle-EOY02487       (1)  -----------MADRDR--PHQIQVHQHHRFDQG--GKNYQSASGP----SATQVLAVLTLLPVGGILLALAGLTLTGTVIGLCVATPLFIIFSPVLVPA
FpuOle-ABQ57396       (1)  -----------MADRPQ--AHQIQVHPQYGKG----FQQRGEQQGP----SAGKILAVLTLLPVGGTLLFLAGLTFIGTLIGLALSTPVFILFSPVLVPA
PpeOle-EMJ16302       (1)  -----------MADRPQ--PHQLQVRPQHG------LGAKTQYQGP----STGQVLAVITGLPVGGTLLALAGLTLMGTIIGALLATPLFIIFSPVLVPA
JcuOle-ABW90149       (1)  -----------MAERSQ--PHQVQVHPQHRYE----AAFKGQQKGP----SAQKVLAVITLLPVGGGLLALAGITLVGTLIGLAITTPLFVIFSPVLVPA
VfoOle2-GU245885      (1)  -----------MAERLQ--PHQVQVHPQLRYD----AYK-GQQKRP----STSKVLAVITLLPLGGGLLALSGITLVGTLIGLAITTPLFFIFSPVLVPA
PtrOle-XP_002300049   (1)  -----------MADRMQ--PHQVQVH----------GLKGHQQQGP----SASKALAVLTMLPLGGGLLALAGITLVGTLIGLAVTTPLFILFSPVLVPA
PtrOle-XP_002323804   (1)  -----------MADRKQ--PHQVQVH----------GLRGQQQQGP----SASKVLAVLTMLPVGGGLLALAGITLVGTLIGLTVTTPLFFLFSPVLVPA
CcaOle-AAX49391       (1)  -----------MADRDRSPPQQLQVHHQHLGYGEGGVKTLFPQTGP----SAIQVLAVVTLLPVGGTLFGLAGLTLVGTLIGLALTTPLFVICSPVLVPA
ColOle-ABF57561       (1)  -------------MADR--PHQIQVRPHHRYDGG--LKTRQDG--P----SPSKVLAVMALLPLGGTLLCLAGITLVGTLIGLAVTTPLFIICSPVLIPA
ColOle-ABF57562       (1)  -----------MADRPY--PHQIQVHPQYSYDSV--IKSLYPQRGP----SPSKVLAILALP-VSGTLLGLSGISLAGSLIGLAVVTPLFFIFSPVLVPA
Consensus             (1)                                               P     S QVLA LT L VGGSLLVLSGLTL GTVIGL VATPLLVIFSPVLVPA

                           101                                                                                                200
CarOle-AAY14574      (75)  VFALGLALAG--FLTSGAFGITALASLSWMLNYIRLMKASS------QEQMDLAKWRVQDTAGQVGQKAR-----------DVGQRTQDVARA-------
CcaOle-AAX49389      (75)  VFTLGLTLAG--FLTSGAFGITALASLSWMLNYIRLMKASS------QEQMDLAKWRVQDTAGQVGQKAR-----------DVGQRTQDVARA-------
ColOle-ABF57559      (77)  ALTVALAVAG--FLTSGAFGITALSSLSWIINYIRRSRMP--------EPMEYAKRRVHDAAGQMGQKTR-----------EVGQKAQESVRT-------
VviOle-XP_002281381  (67)  ALVLALAITG--FLTSGAFGITALSSLSWIVNYLRKGRVT--------EQMEHARWRVQEGAGQLGMKAK-----------EVGHGIQGKAQETTRT---
JcuOle-ABW90148      (74)  ALVIGLSVLG--FLTSGAFGITALSSLSWMVNY--IRRMRG----SWTMQMEMAKRRAQETTGQLGQKAR-----------EVGQKAQEVAKT-------
RcoOle-XP_002511342  (69)  AFVIGLAVLG--FSVSGAFGITALSSLSWMLNN--FRRMIG----LLPQQMEHAKRRVQETTGQLGQKAR-----------DVGQTVQSKAQ--------
VfoOle3-GR217899.1   (82)  AFVIGLSVMETGFLASGAFGISALSSLSWMETVNYIRRMETRGSLPLQMETEHAKRRVQDTTGQLGQKARDM---------ETGQTVQSKAQEGGKT---
PtrOle-XP_002318659  (72)  ALVIGLGVLG--FLASGAFGVTALSSLSWMASH--SQSLIRG---PLTQTLDQAKRKAQETAGQLGQKAR-----------ETGQTVQ------------
PtrOlep-XP_002322186 (27)  ALVIGLGVLG--FLTSGAFGVTALSSLSWMASY--IRSLIRG---PLPQKLDQAKRRTRETAGQVGQKAR-----------ETGQIVQSKARE-------
PpeOle-EMJ10773      (97)  ALVIGLSVVG--ILTSGAFGITALSSFSWLARFLSRSRLPQKMGQKVQETTGYLGLKVQETAGYLGQKMQETGGQVGHLLQETGGQVGQKTRETGQNVDK
CavOle-AAO65960      (66)  VITVSLIIMG--FLASGGFGVAAVTVLSWIYRYVTGRHPPG------ADQLDHARMKLASKAREMKDRAE-----------QFGQQHVTGSQGS------
JreOle-AET74076      (66)  VITVCLIIFG--FLASGGFGVAALTVLSWIYRYMTGRQPPG------TEQIDQARMKLASKAREMKDKAE-----------QFGQQHVSGAQSS------
JreOle-AET74077      (66)  VITVALIILG--FLASGGFGVAAVTVLSWIYRYVTGRQPPG------AEQIDQARMRLASKAREMKDRAE-----------QFGQQHVSGAQG-------
ColOle-ABF57560      (66)  AIAVCLLILG--FVASGGFGLAAVSVLSWIYRYVTGKQPPG------AEQLDYARMKLASKAREMKDKAE-----------LFGQQHVTGSQQAW-----
VviOle-XP_002281746  (71)  AIAVFFLVLG--FLASGGFGVAAVTVLSWIYRYVTGRHPPG------ADHLDSARMKLASKAREMKDRAE-----------QFGQQHTG--QQGT-----
CcaOle-AAX49390      (60)  GITVFLLVTG--FLSSGGFGVAALSVLSWIYGYVTGKNPPG------ADQLDRARQKLALKAREMKDRAE-----------QYGQQNIPAGSQQH-----
ColOle-ABF57563      (66)  AVTVCLLNMG--FLASGGFGVAGISVLSWIYRYVTGKHPPG------ADQLDQARTKLASKAREMKDRAE-----------QFGQQHIS-GSQAS-----
FpuOle-ABQ57397      (77)  VITLGLIIIG--FLASGGFGVAALTVLSWIYRYVTGKHPPG------ADQLDQARHKLASKAREMKDKAE-----------QFGQQHLTSGQQQSS----
PduOle-Q43804.1      (72)  LITVALITMG--FLTSGGFGVAAVTVLSWIYKYVTGKQPPG------ADQLDQARHKLAGKARDIKDRAE-----------QFGQQHVPSGQQQSS----
PpeOle-EMJ19764      (72)  LITVALITMG--FLTSGGFGVAAVTVLSWIYKYVTGKQPPG------ADQLDQARHKLAGKARDIKDRAE-----------QFGQQHVPSGQQQSS----
CsiOle-CAA88360      (69)  VITVSLLIMG--FLASGGFGVAAISVLSWIYRYVTGGHPPG------ADQLEQARMKLAGKAREMRDRAE-----------QFGQQQITGSQQGS-----
PtrOle-XP_002297927  (65)  VMAVSLLLMG--FLASGGFGVAGITAMSWIYGYVTGRHPPG------SDQLEQARIKLAVKAREMKDRAE-----------QFGHQVIS-----------
```

```
   PtrOle-XP_002304591  (65)  VITIYLLLMG--FLASGGFGVTGITVMSWMYRYVTGRHPPG------AEQLDQAGMKLVGKAREMKERGE-----------QFGLQAAQ-----------
      JcuOle-ABW90150   (65)  VITVCLIITG--FLASGGFGVAAIFVLFWIYRYVTGKHPPG------AENLDQARLKLAGKAREMKDRAE-----------QFGQHVTGQQT--------
      JcuOle-AFP19885   (65)  VITVCLIITG--FLASGGFGVAAISVLSWIYRYVTGKHPPG------AESLDQARLKLAGKAREMKDRAE-----------QFGQHVTGQQT--------
    VfoOle1-GU245884    (65)  VITVFLIVTG--FLASGGFGVAAISVLSWIYRYVTGKQPPG------AESLDQARMKLAGKAREMKDRAE-----------QLGQHVTGQHT--------
   RcoOle-XP_002511014  (65)  VIIISLIGAG--FLTSGGFGFGAILVLSWIYRYVTGKQPPG------AESLDQARLKLAGKAREMKDRAE-----------QFGQHVTGQQTS-------
      TcaOle-EOY22772   (69)  LIAAALLVTG--FMASGGFGVAAITVLSWIYRYVTGGHPPG------ADQLDQARMTLARKAREMNEGPE-----------QLTSAAAS-----------
    EguOlep-AF273023_1  (70)  VIAVFLLVTG--FVTSGGLGVAALSVLSWLYKYLTGKRVPG------SEQLEQARARLASKARDVKESAQ-----------HRSEQAQTS----------
      CcaOle-AAX49392   (63)  AVTFFLILAG--FFISGGLGVTATFIFYWMFRYATGKHPIG------ADQLDRAREKIAHAAKEMRDKAE-----------HFGQQAQQQIKGSPDQDT-
   VviOle-XP_003631370  (62)  AITVFLAASG--LVASGGFGVSAVSVFLWLYKYVRGQHPVG------ADRLDRARDKLTGKAMEVKERAA-----------EQIGTRGTQG---------
      PamOle-AGT63296   (66)  --T-FLVATG--FIFSSGFGIAALSMVAWMYRYATGKHPPG------LEQLDHVMKVLVHEAKIMKNKAK-----------EYAYQVRDMAQQITKRVQN
      PpeOle-EMJ25876   (74)  GIVVFLTAAG--LVFSGGCGVAAVTTLTLMYKYISSYVATK------KRAYSYGQYFCPF----------------------------------------
      PpeOle-EMJ26259   (59)  GIVMLLIAAG--FVFSGGCGMAAMTVMAWLYNYVSNYAAAK------QRANAYRQFVMLDI---------------------------------------
   PtrOle-XP_002309527  (69)  AIVVFLVASG--FFFSSGCGLAAIMVSLWIYNYVTGKHPPG------ADKLDYAGGRIAETAKDMKDRAK-----------ECGQNVRQKVQESS-HTQT
   PtrOle-XP_002334246  (63)  AMVVFLVSSG--FFFSGGCGLAAIMVLLWTYNYVTGKHPPG------ADRLDYATRKIAEKAK------------------EYGQYVQPKAQEAT-QTS-
    VfoOle5-GR218198    (68)  GIVVFLVATG--FFFSGGCGLAAIMALTWMYNYLTGKHPPG------ADKLDYARGKIAKKAQDMKE------------------YVQQKAQEATTQTRA
      TcaOle-EOY29822   (69)  GVVIFLVITG--FLFSGGCGVAAITALSWIYNYVRGKHPPG------ADQLDYARNTLARTARDMTEKAK-----------EYGQYVQHKAQEVAQGS--
   VviOle-XP_002275496  (68)  GTVIFLATTG--FLFSGCCGVTALMALSWLYEYVAGKHPPG------ADQLDYARMRIANKARDMKERAK-----------EYGQYVHHKAQEATQGS--
      CcaOle-AAX49393   (89)  GSILFVIIAG--LLSFFGFGVAAMAAFSWLYRYFRGFHP------PGSDRVDYARSRIANTASQVKDYAR-----------EYGGYLHGKVKDAAPGA--
      PpeOle-EMJ10882   (96)  GALLFLTVAG--FVSMCGFGAAAAAVSSWMYRYFKGMHP------PGSDRVDYARSRIYDTASHVKDYAR-----------EYGGYLHSKVKDAAPGA--
      TcaOle-EOY20922   (85)  GTVLFVTVAG--FLSACGFGLVVMAALSWMYRYFRGMHP------PGSDRVDYARSRIYDTASHVKDYAM-----------EYGGYLQSKVKDAAPGA--
   PtrOle-XP_002317928  (86)  GIVLFFVVAG--FLSFCGFGLAVVAGLSWVYKYFRGLNP------PGSDQVDYARNRIYDTASHVKDYAR-----------EYGGYLQSKVKDAAPGA--
   RcoOle-XP_002511984  (91)  GVLLFFAAAG--FFSVCGFLVATIGGLSWMYRYYRGMHP------PGSDRFDYARSRIYDTASHVKDYAR-----------EYGGYLQSKVKDAAPGA--
              VfoOle4   (89)  AVILFISVAG--FLSICGFVAAVVGGLSWAYRYFRGMETNP----PGSDQFDYARSRIYDTASHVKDYAR-----------EYGGYLQSKVKDAAPGA--
      VviOle-CBI15408   (83)  GTVLFLAAAG--FLSMCGFGLAVLAGLSWIYKYFRGWNP------PGSGRFDYARSRIADTASHVKDYAR-----------EYGGNPTAGIYPPSGSPSA
   VviOle-XP_002273242  (83)  GTVLFLAAAG--FLSMCGFGLAVLAGLSWIYKYFRGWNP------PGSGRFDYARSRIADTASHVKDYAR-----------EYGGYLQSKVKDAAPGA--
      PtaOle-AFG55850   (24)  ATVLFVAVAG--FLSAGGFGLAALSAISWLYNYIKGRHP------PGADQIDYARMRIADTASHVKDYAR-----------EYGGYLQSKIQDAAPGA--
      PtaOle-AFG55851   (24)  AIVLFVAVAG--FLSAGGFGLAALSAISWLYNYIKGRHP------PGADQIDYARMRIADTASHVKDYAR-----------EYGGYLQSKIQDAAPGA--
      TcaOle-EOY20852   (81)  ALVIAGSVAG--FLTSGAFGITGLSSLSWIVNYLRGTRGSM------SQRLDHAKRRSFLCYLLCFQLLACKN----QYKVGEGQEGYTKCMKTNQMNAG
   VviOle-XP_002273158  (61)  AIGIGLAVTG--FLSSGALGVTALSSLSWFLNYLRQLAAG-------------MPDYFQRVTDKLGRETK-----------EMGQELQSQAHEGWGKPTK
   VviOle-XP_002275087  (73)  AVVVGLAVSG--FITSQTLGATGVSALSCAWDYLQRVLESSP-LKGVVDRMGKGHALKDGPVSLVQFET-------------------------------
   VviOle-XP_002275021  (82)  TIAIGLAVVS--FVTSGAFGVTGLSSLSFAVNSFRQAAAP-------------LPGYLRSAMDKLGEKTK-----------GLGQGIQKQAHEGG--PIR
      OeuOle-AAL92479   (86)  TILVGLAVTA--FLTSGAFGLTGLSSLSWVVNFLRQVSG---------SMLDLAKSRMGDAAIQVGQKTK-----------ETGQTIQQKAPEGKESTGG
      CavOle-AAO67349   (82)  AIVVGLAVAS--FLSSGALGLTGLSSLSWVLNYLRCASQSL------PREMDQAKRRMQDMAAFVGQKTR-----------EVGQEIQSRAQEGRRT---
      TcaOle-EOY02487   (82)  AIAVGLAVAG--FLSSGAFGLTGLSSLAYVFNRLRRATGTE------QLDMDQAKRRMQDMAGYVGQKTK-----------EVGQKIEGKANEGTVRT--
      FpuOle-ABQ57396   (80)  AITIGLAITG--FLTSGAFGVTGLSSLSWVFNYFKRTS--V------PDQLDYAKRRVQDMAGYTGQKAK-----------EVGQEVQSKGQEGKRT---
      PpeOle-EMJ16302   (78)  IFVIGLAVTG--FLTSGAFGLTALSSLSWVTNYLRRATGLV------PEQLDQMKRRTADMVEFVGQKTK-----------EGSQDIQSKAQDEKRRT--
      JcuOle-ABW90149   (80)  ALVIGLSVMA--FLASGAMGLTGLSSLSWVLKYLQEVTRRM------PEQLDIAKKRMQDMAGFVGQKTK-----------EVGQEIQRKAHEGK-----
    VfoOle2-GU245885    (79)  AFVLGLAVMT--FLASGAMGLTGLTSLSWVLKYVQEATRNI------PEQLDQARRRMQDMAGYVGQKTK-----------EMGQEIQKKTHEGK-----
   PtrOle-XP_002300049  (74)  ALVIGLAVTS--FLASGAFGLTGSWSLSWVGRYIQEATQTM------PESLDQAKRRMQDMAGYVGQKTK-----------EVGQEIQRKAHDGK-----
   PtrOle-XP_002323804  (74)  ALLIGFAATS--FLASGALGLTGFRSLSWVARYVQEATRTM------PENLDQAKRCMQDMAGYVGQRAK-----------EVGQEIQRKAHEGK-----
      CcaOle-AAX49391   (86)  VVLFGLAVTG--FLSSGAFGLMGLSSLSWVLNCFRQKAT---------EQMDDARRRMQEAAGQLGQKTK-----------EVGERIQTKAQEPTGRDQG
      ColOle-ABF57561   (78)  AIATGLAVAG--ILTSGAFGLTGLSWLTRVVNYLRGVGASV------PQQLDEAKRRMQDVAVQMGEKTK-----------EVGEAIQKKAQEAGK----
      ColOle-ABF57562   (81)  AIVACLTLAG--LMASGAFGLTGLTAMSWVLNYLRQASAGM------PQQMDEVKRRMQDVAIPMGQKTK-----------EVGETIHKKAQEAGQ----
            Consensus  (101)  AIVV L V G  FLASGGFGVAALSSLSWIYRYV G  P        ADQLD AR RL D A  MKDKAK           E GQ VQ  A
                              201                                                                                               300
      PpeOle-EMJ10773  (195)  AQDAGRDQEGGRTKEGGRGREGVTVTVEP-----------------------------------------------------------------------
      PamOle-AGT63296  (144)  MGNSEILFSENRLKVF------------------------------------------------------------------------------------
   PtrOle-XP_002309527 (149)  S---------------------------------------------------------------------------------------------------
     VfoOle5-GR218198  (142)  S---------------------------------------------------------------------------------------------------
      VviOle-CBI15408  (164)  TRRRLRTLPLQLLCCLTVRPLSSYAEKESPCCLYSSAAAWAAPTATSERFSSGSPLYETCWKLGWLAWSTKPQASIRSGASHLRPSWRFQSRCWSRCRVL
      TcaOle-EOY20852  (169)  GKTKQNE---------------------------------------------------------------------------------------------
   VviOle-XP_002273158 (135)  RIQIGL----------------------------------------------------------------------------------------------
   VviOle-XP_002275021 (154)  KLGY------------------------------------------------------------------------------------------------
      OeuOle-AAL92479  (164)  RT--------------------------------------------------------------------------------------------------
      CcaOle-AAX49391  (164)  VREAGKYVEKFQ----------------------------------------------------------------------------------------
                              301                                                    355
      VviOle-CBI15408  (264)  GYGGPWLRAVNGFIGREGGRRSSSNCAGYDWSTYKTQTLGRKALDHGFHLSCPAS
```

Figure S2. Multiple sequence alignment for the identification of amino acid residues and sequence motifs conserved in OLE. Multiple sequence alignment was performed using the ClustalW algorithm of the AlignX program of the Vector NTI software. Each OLE sequence name is on the left of the alignment followed by the position of amino acid residue of OLE protein sequence in the alignment. The letters at the bottom of the alignment are the consensus residues. Color codes for amino acid residues are as follows: 1) red on yellow: consensus residue derived from a completely conserved residue at a given position; 2) black on green: consensus residue derived from the occurrence of greater than 50% of a single residue at a given position; 3) blue on cyan: consensus residue derived from a block of similar residues at a given position; 4) green on white: residue weakly similar to consensus residue at a given position; 5) black on white: non-similar residues. The abbreviations of the organisms are: Car, *Coffea arabica* (coffee); Cca, *Coffea canephora* (coffee); Cav, *Corylus avellana* (hazelnut); Col, *Camellia oleifera* (tea oil); *Citrus sinensis* (orange); Egu, *Elaeis guineensis* (oil palm); Fpu, *Ficus pumila* (climbing fig); Jcu, *Jatropha curcas* (barbados nut); Jre, *Juglans regia* (walnut); Oeu, *Olea europaea* (olive); Pam, *Persea Americana* (avocado); Pdu, *Prunus dulcis* (almond); Ppe, *Prunus persica* (peach); Pta, *Pinus taeda* (loblolly pine); Ptr, *Populus trichocarpa* (poplar); Rco, *Ricinus communis* (castor bean); Tca, *Theobroma cacao* (cacao); Vfo, *Vernicia fordii* (tung tree); Vvi, *Vitis vinifera* (grapevine).
