# Supplementary material for: Identification, Classification and Differential Expression of Oleosin Genes in Tung Tree (Vernicia fordii)
Source: PLoS One. 2014 Feb 6;9(2):e88409. doi: 10.1371/journal.pone.0088409 (PMC3916434; doi:10.1371/journal.pone.0088409)
Supplement: Figure S3 — Specificity of SYBR Green qPCR Assay. The qPCR reactions contained 5 ng RNA-equivalent cDNA from tung tree leaves and flowers. The qPCR products were separated by agarose gel electrophoresis. Lane 100 bp represents DNA ladders with 100 bp as the smallest band, increasing upward in 100 bp increments. The results using tung tree seeds are shown in Figure 3B. (A) Melt curve analysis, (B) Gel electrophoresis. (PDF) [file pone.0088409.s003.pdf]

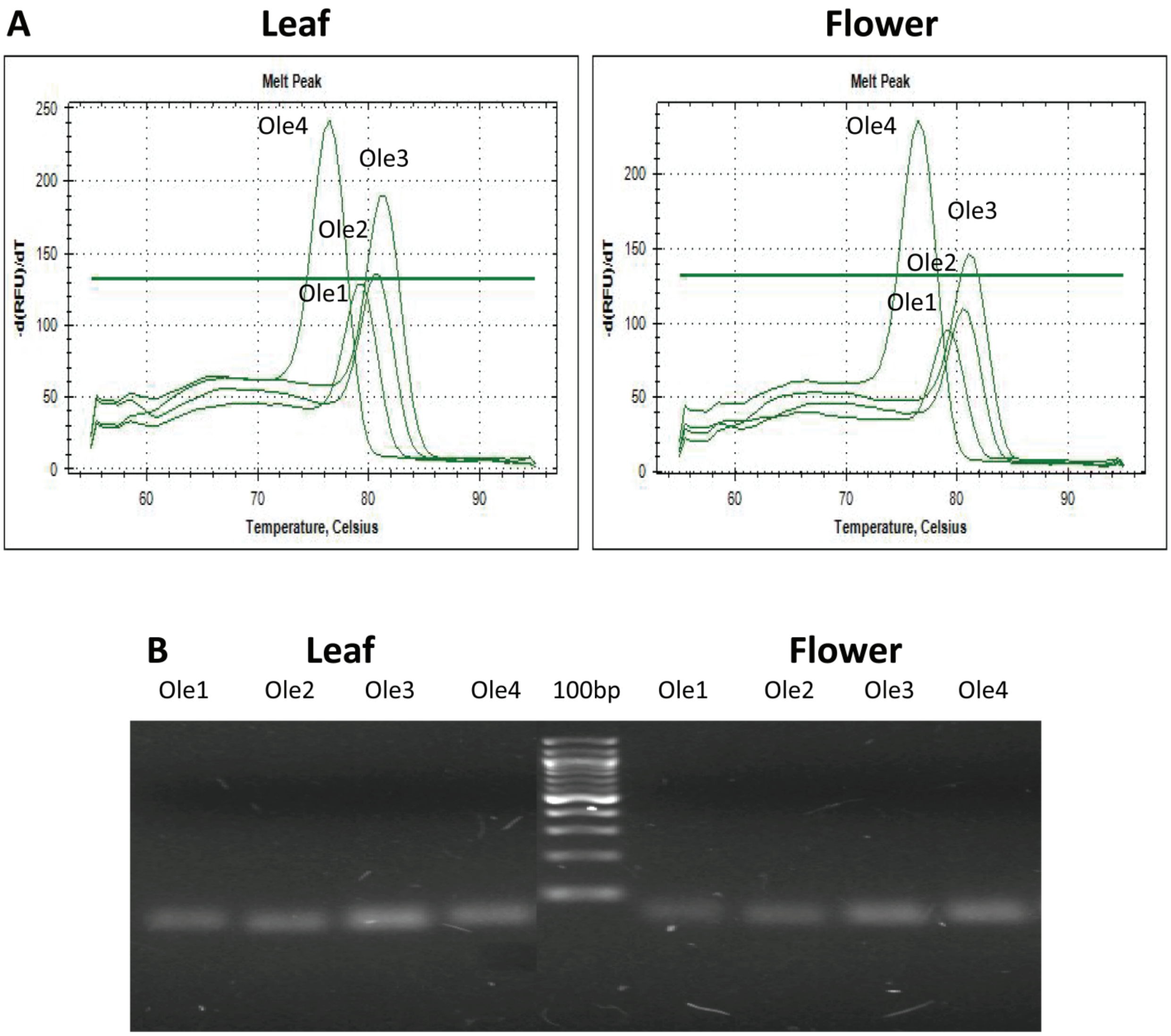

Figure S3. Specificity of SYBR Green qPCR Assay. The qPCR reactions contained 5 ng RNA-equivalent cDNA from tung tree leaves and flowers. The qPCR products were separated by agarose gel electrophoresis. Lane 100bp represents DNA ladders with 100 bp as the smallest band, increasing upward in 100 bp increments. The results using tung tree seeds are shown in Figure 4B. (A) Melt curve analysis, (B) Gel electrophoresis.
