# Supplementary material for: Identification, Classification and Differential Expression of Oleosin Genes in Tung Tree (Vernicia fordii)
Source: PLoS One. 2014 Feb 6;9(2):e88409. doi: 10.1371/journal.pone.0088409 (PMC3916434; doi:10.1371/journal.pone.0088409)
Supplement: Figure S4 — qPCR efficiency for OLE assay. TaqMan and SYBR Green qPCR reaction mixtures contained variable concentrations of RNA-equivalent cDNA from tung seeds, the optimized concentrations of each primer and probe (200 nM), and Absolute QPCR Mix (TaqMan qPCR) or each primer and 1 x iQ SYBR Green Supermix (SYBR Green qPCR). The results using RNA isolated from stage 4 seed of tree 1 are shown. The qPCR efficiency for Ole1 mRNA detection is presented in Figure 3C. The results using RNA from other stages of tung seeds, leaves and flowers are presented in Table S1. (A) qPCR efficiency for Ole1 mRNA detection, (B) qPCR efficiency for Ole2 mRNA detection, (C) qPCR efficiency for Ole3 mRNA detection, (D) qPCR efficiency for Ole4 mRNA detection. (PDF) [file pone.0088409.s004.pdf]

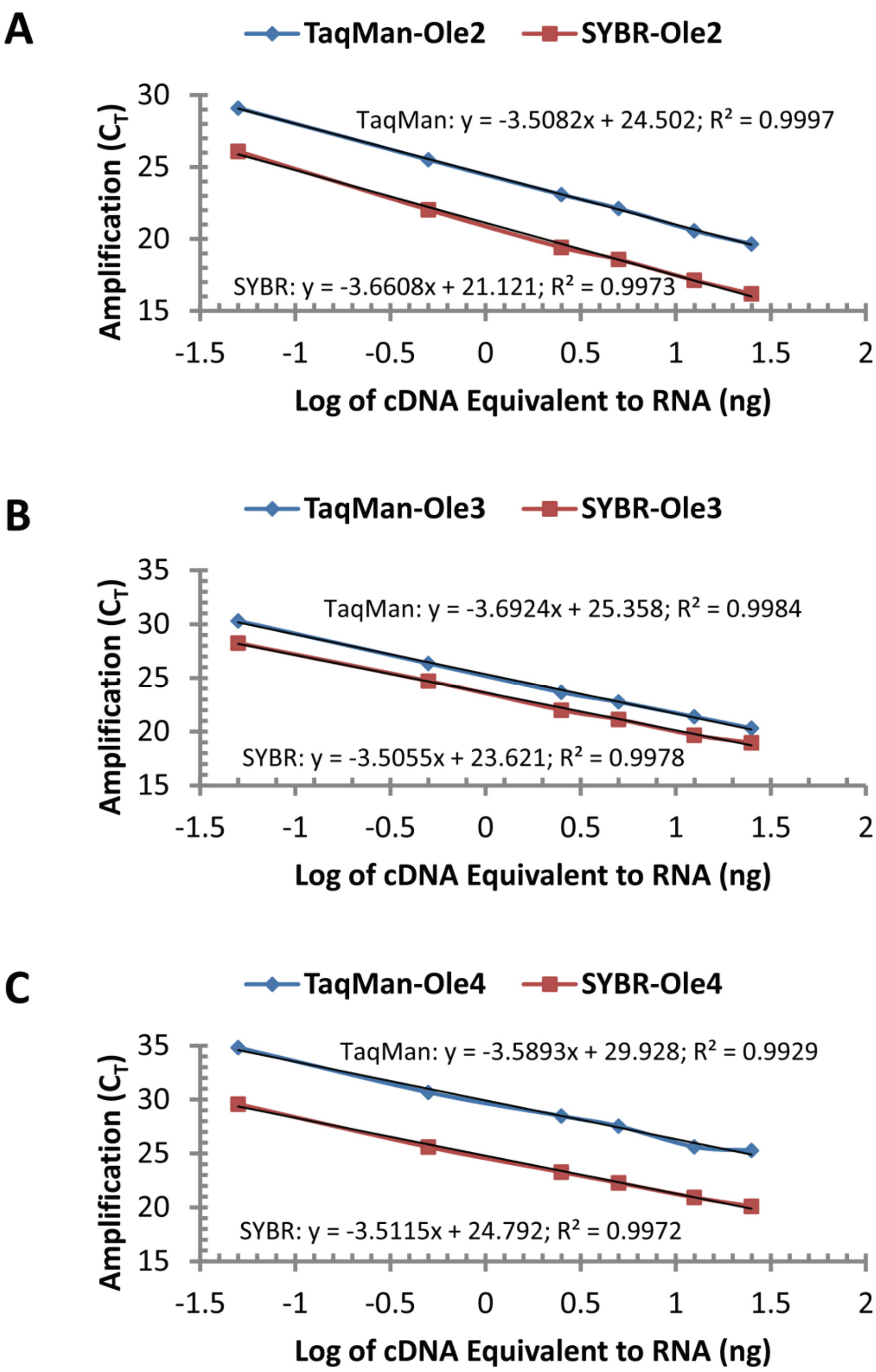

Figure S4. qPCR efficiency for OLE assay. TaqMan and SYBR Green qPCR reaction mixtures contained variable concentrations of RNA-equivalent cDNA from tung seeds, the optimized concentrations of each primer and probe (200 nM), and Absolute QPCR Mix (TaqMan qPCR) or each primer and 1 x iQ SYBR Green Supermix (SYBR Green qPCR). The results using RNA isolated from stage 4 seed of tree 1 are shown. The qPCR efficiency for Ole1 mRNA detection are presented in Figure 4C. The results using RNA from other stages of tung seeds, leaves and flowers are presented in Table S1. (A) qPCR efficiency for Ole2 mRNA detection, (B) qPCR efficiency for Ole3 mRNA detection, (C) qPCR efficiency for Ole4 mRNA detection.
