# Supplementary material for: Identification, Classification and Differential Expression of Oleosin Genes in Tung Tree (Vernicia fordii)
Source: PLoS One. 2014 Feb 6;9(2):e88409. doi: 10.1371/journal.pone.0088409 (PMC3916434; doi:10.1371/journal.pone.0088409)
Supplement: Figure S5 — Relative levels of OLE gene expression in developing tung seeds by TaqMan qPCR. The qPCR reaction mixtures contained 25 ng of RNA-equivalent cDNA from tung seeds and 200 nM of each primer and probe. The means of mRNA expression levels calculated from two qPCR assays in each seed stage using Rpl19b as the reference mRNA are presented in Figure 4A. The means of mRNA expression levels calculated from two qPCR assays in each seed stage are presented. (A) Gapdh as the reference mRNA, (B) Ubl as the reference mRNA. (PDF) [file pone.0088409.s005.pdf]

A

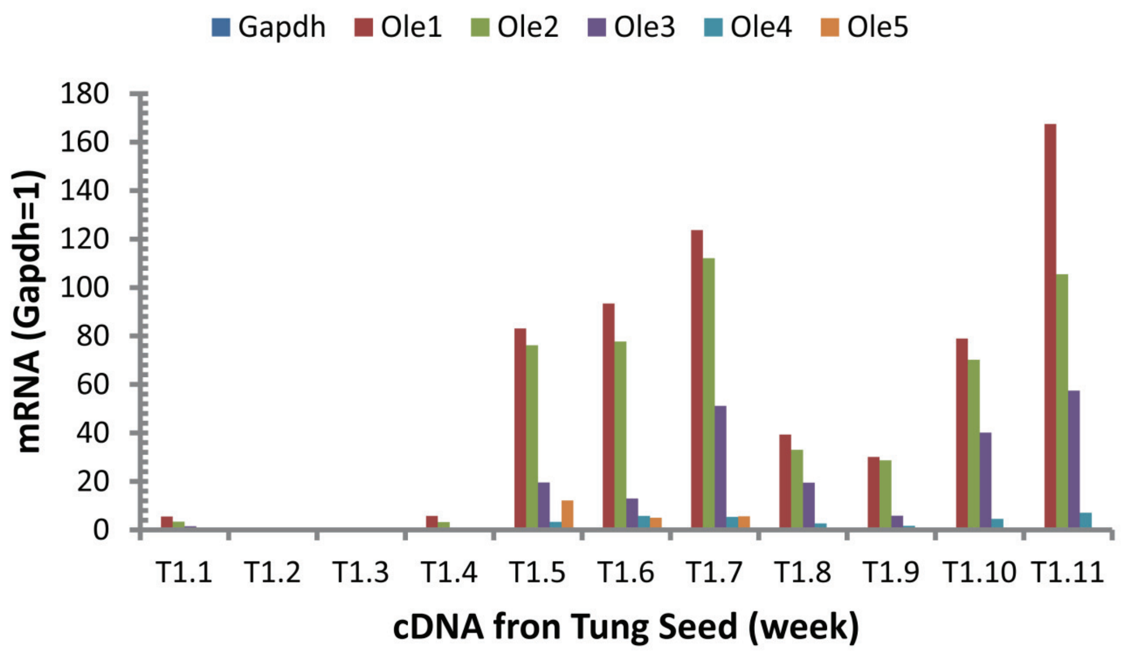

B

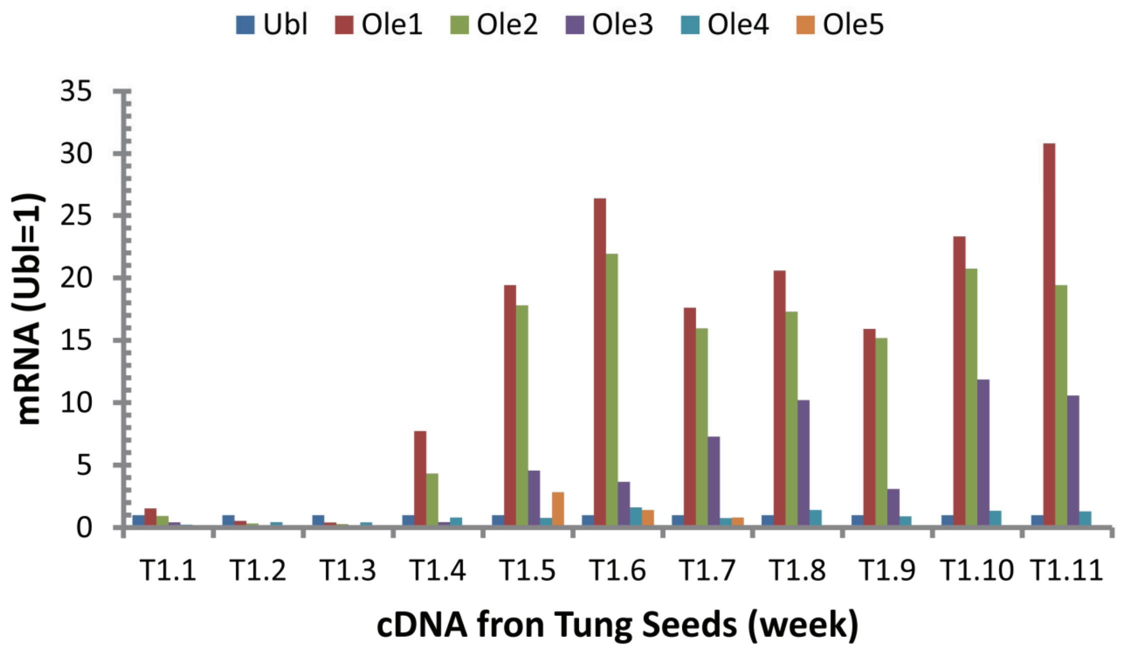

Figure S5. Relative levels of OLE gene expression in developing tung seeds by TaqMan qPCR. The qPCR reaction mixtures contained 25 ng of RNA-equivalent cDNA from tung seeds and 200 nM of each primer and probe. The means of mRNA expression levels calculated from two qPCR assays in each seed stage using Rpl19b as the reference mRNA are presented in Figure 5A.The means of mRNA expression levels calculated from two qPCR assays in each seed stage are presented. (A) Gapdh as the reference mRNA, (B) Ubl as the reference mRNA.
