# Supplementary material for: Identification, Classification and Differential Expression of Oleosin Genes in Tung Tree (Vernicia fordii)
Source: PLoS One. 2014 Feb 6;9(2):e88409. doi: 10.1371/journal.pone.0088409 (PMC3916434; doi:10.1371/journal.pone.0088409)
Supplement: Figure S6 — Relative levels of OLE gene expression in tung tissues by SYBR Green qPCR. The qPCR reaction mixtures contained 5 ng of RNA-equivalent cDNA from various stages of tung seed, leaves and flowers and 200 nM of each primer. The means of mRNA expression levels calculated from two qPCR assays in each seed stage using Rpl19b as the reference mRNA are presented in Figure 4B. The means of mRNA expression levels calculated from two qPCR assays in each seed stage are presented. (A) Gapdh as the reference mRNA, (B) Ubl as the reference mRNA. (PDF) [file pone.0088409.s006.pdf]

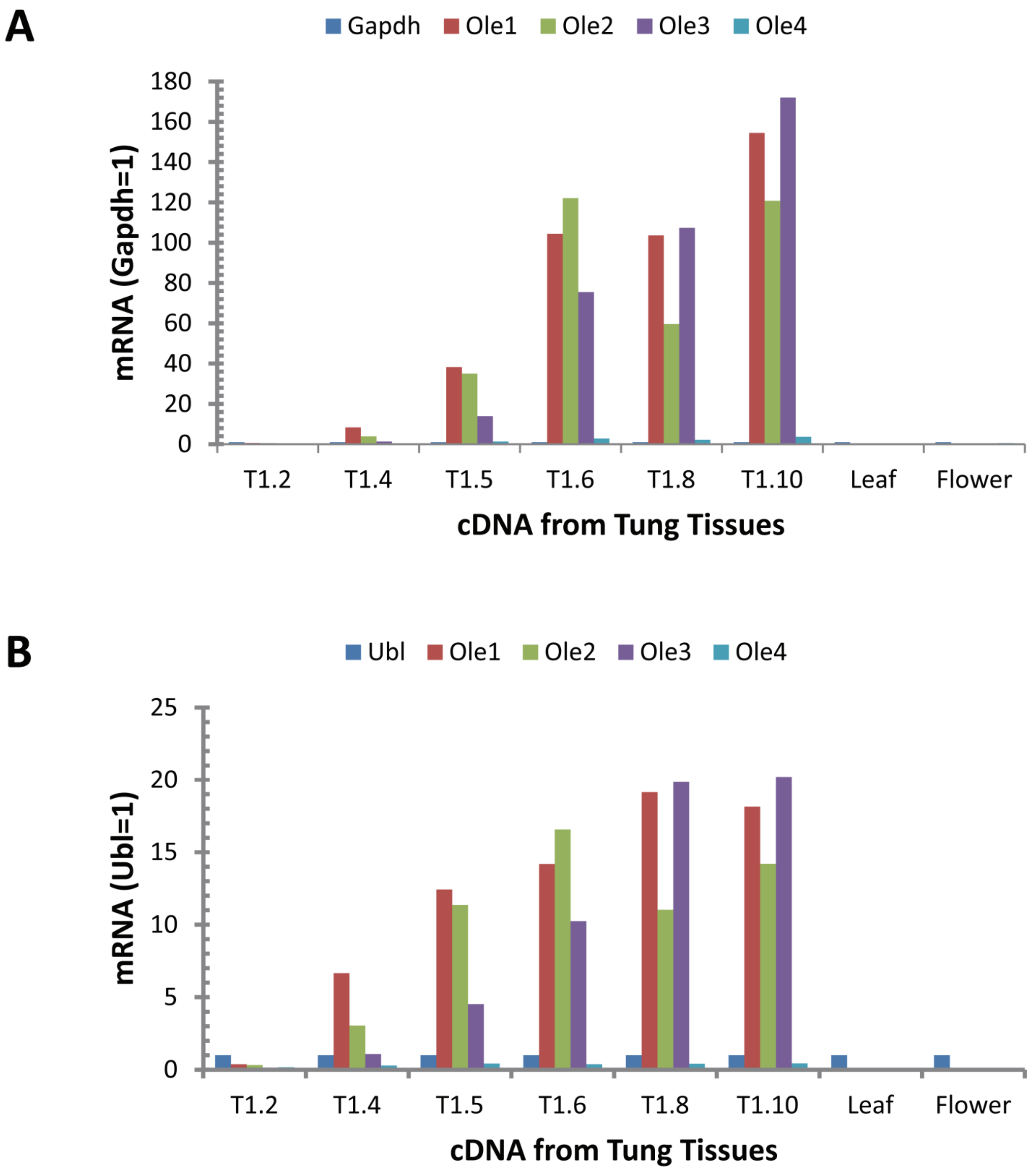

Figure S6. Relative levels of OLE gene expression in tung tissues by SYBR Green qPCR. The qPCR reaction mixtures contained 5 ng of RNA-equivalent cDNA from various stages of tung seed, leaves and flowers and 200 nM of each primer. The means of mRNA expression levels calculated from two qPCR assays in each seed stage using Rpl19b as the reference mRNA are presented in Figure 5A.The means of mRNA expression levels calculated from two qPCR assays in each seed stage are presented. (A) Gapdh as the reference mRNA, (B) Ubl as the reference mRNA.
