# Supplementary material for: Identification, Classification and Differential Expression of Oleosin Genes in Tung Tree (Vernicia fordii)
Source: PLoS One. 2014 Feb 6;9(2):e88409. doi: 10.1371/journal.pone.0088409 (PMC3916434; doi:10.1371/journal.pone.0088409)
Supplement: Table S1 — TaqMan qPCR efficiency for quantifying Ole mRNA in tung tree tissues. (PDF) [file pone.0088409.s007.pdf]

Table S1. TaqMan qPCR efficiency for quantifying Ole mRNA in tung tree tissues.

Tung tissue	mRNA	Slope	Y-Intercept	Correlation Co-efficiency	Efficiency E=[10(1/-S)-1]*100 (%)
Seed (2 weeks)	Ole1	-3.3962	26.861	0.9998	97
	Ole2	-3.6044	27.547	0.9986	89
	Ole3	-3.8020	27.912	0.9978	83
	Ole4	-3.4817	29.681	0.9997	94
Seed (4 weeks)	Ole1	-3.4709	23.897	0.9997	94
	Ole2	-3.5082	24.502	0.9997	93
	Ole3	-3.6924	25.358	0.9984	87
	Ole4	-3.3893	29.928	0.9929	90
Seed (6 weeks)	Ole1	-3.4313	23.272	0.9994	95
	Ole2	-3.6393	23.364	0.9994	88
	Ole3	-3.5800	23.707	0.9983	90
	Ole4	-3.6150	30.064	0.9898	89
Seed (10 weeks)	Ole1	-3.3300	22.220	0.9992	100
	Ole2	-3.4564	22.545	0.9991	95
	Ole3	-3.5145	20.878	0.9990	93
	Ole4	-3.4344	29.110	0.9967	95
Leaf	Ole1	-3.4126	35.856	0.9958	96
	Ole2	-3.1539	34.505	0.9959	107
	Ole3	-3.4722	34.761	0.9841	94
	Ole4	-3.6923	34.791	0.9866	87
Flower	Ole1	-3.0942	37.921	0.9831	110
	Ole2	-3.5341	35.983	0.9982	92
	Ole3	-3.4672	31.241	0.9997	94
	Ole4	-3.4919	31.982	0.9970	93

qPCR reaction mixtures contained variable amounts of RNA-equivalent cDNA from tung seed (0.05, 0.5, 2.5, 5, 12.5 and 25 ng), the optimized concentrations of each primer (200 nM) and probe (200 nM) and qPCR Mix.
