# Supplementary material for: Identification, Classification and Differential Expression of Oleosin Genes in Tung Tree (Vernicia fordii)
Source: PLoS One. 2014 Feb 6;9(2):e88409. doi: 10.1371/journal.pone.0088409 (PMC3916434; doi:10.1371/journal.pone.0088409)
Supplement: Table S2 — Variation of Ole gene expression among tung trees. (PDF) [file pone.0088409.s008.pdf]

Table S2. Variation of Ole gene expression among tung trees by SYBR Green qPCR assay.

mRNA	Tree 1 (fold)	Tree 2 (fold)	Tree 3 (fold)	Mean ± SD (fold)
Ole1	1	0.81 ± 0.17	0.88 ± 0.17	0.90 ± 0.10
Ole2	1	0.68 ± 0.15	0.52 ± 0.10	0.73 ± 0.24
Ole3	1	0.74 ± 0.16	0.47 ± 0.09	0.74 ± 0.26
Ole4	1	0.64 ± 0.14	0.60 ± 0.11	0.75 ± 0.22

The SYBR Green qPCR reaction mixtures (12.5 μl) contained 5 ng of RNA-equivalent cDNA from various stages of tung seeds, the optimized concentrations of each primer (200 nM) and QPCR Mix. The expression levels under each tree represent the means and standard deviations of the expression fold calculated using three reference mRNA (Rpl19b, Gapdh and Ubl) from 6 stages of seeds (weeks 2, 4, 5, 6, 8 and 10) with 2-4 assays for each stage. Ole gene expression in tree 1 seeds was used as the calibrator for the calculation of Ole gene expression in tree 2 and tree 3 seeds.
