# Supplementary material for: Identification, Classification and Differential Expression of Oleosin Genes in Tung Tree (Vernicia fordii)
Source: PLoS One. 2014 Feb 6;9(2):e88409. doi: 10.1371/journal.pone.0088409 (PMC3916434; doi:10.1371/journal.pone.0088409)
Supplement: Table S3 — Ole gene expression among different stages of tung seeds. (PDF) [file pone.0088409.s009.pdf]

Table S3. Ole gene expression among different stages of tung seeds.

qPCR method	mRNA	2 (fold)	4 (fold)	6 (fold)	10 (fold)
TaqMan	Ole1	1	11.05 ± 2.49	39.58 ± 18.26	97.29 ± 74.92
	Ole2	1	9.40 ± 2.12	48.38 ± 22.32	109.67 ± 84.45
	Ole3	1	6.77 ± 1.33	40.91 ± 2.19	413.10 ± 2.61
	Ole4	1	1.15 ± 0.26	3.11 ± 1.43	6.74 ± 5.198
	Ole5	1	15.63 ± 3.52	17.98 ± 8.30	0.23 ± 0.18
SYBR Green	Ole1	1	14.81 ± 2.66	80.13 ± 63.97	107.53 ± 103.69
	Ole2	1	8.01 ± 1.44	110.56 ± 88.25	99.30 ± 95.75
	Ole3	1	6.17 ± 0.28	148.21 ± 1.45	306.52 ± 1.60
	Ole4	1	1.38 ± 0.25	4.63 ± 3.70	5.58 ± 5.38

The qPCR reaction mixtures contained 25 ng in 25 µl (TaqMan qPCR) or 5 ng in 12.5 µl (SYBR Green qPCR) of RNA-equivalent cDNA from various stages of tung tree 1 seeds, the optimized concentrations of each primer and probe (200 nM) and QPCR Mix. The expression levels under each seed stage represent the means and standard deviations of the expression fold calculated using three reference mRNA (Rpl19b, Gapdh and Ubl) each with 2-4 assays. Ole gene expression in stage 2 seeds was used as the calibrator for the calculation of Ole gene expression in other stage seeds.
